# Epigenetic regulation of human *SOX3* gene expression during early phases of neural differentiation of NT2/D1 cells

**DOI:** 10.1371/journal.pone.0184099

**Published:** 2017-09-08

**Authors:** Vladanka Topalovic, Aleksandar Krstic, Marija Schwirtlich, Diletta Dolfini, Roberto Mantovani, Milena Stevanovic, Marija Mojsin

**Affiliations:** 1 Institute of Molecular Genetics and Genetic Engineering, University of Belgrade, Belgrade, Serbia; 2 Systems Biology Ireland, University College Dublin, Dublin, Ireland; 3 Department of Biosciences, University of Milan, Milan, Italy; 4 Faculty of Biology, University of Belgrade, Belgrade, Serbia; 5 Serbian Academy of Sciences and Arts, Belgrade, Serbia; Beijing Cancer Hospital, CHINA

## Abstract

*Sox3/SOX3* is one of the earliest neural markers in vertebrates. Together with the *Sox1/SOX1* and *Sox2/SOX2* genes it is implicated in the regulation of stem cell identity. In the present study, we performed the first analysis of epigenetic mechanisms (DNA methylation and histone marks) involved in the regulation of the human *SOX3* gene expression during RA-induced neural differentiation of NT2/D1 cells. We show that the promoter of the human *SOX3* gene is extremely hypomethylated both in undifferentiated NT2/D1 cells and during the early phases of RA-induced neural differentiation. By employing chromatin immunoprecipitation, we analyze several histone modifications across different regions of the *SOX3* gene and their dynamics following initiation of differentiation. In the same timeframe we investigate profiles of selected histone marks on the promoters of human S*OX1* and *SOX2* genes. We demonstrate differences in histone signatures of *SOX1*, *SOX2* and *SOX3* genes. Considering the importance of *SOXB1* genes in the process of neural differentiation, the present study contributes to a better understanding of epigenetic mechanisms implicated in the regulation of pluripotency maintenance and commitment towards the neural lineage.

## Introduction

SOX3/Sox3 is an X-linked member of SOXB1 (SOX1-3) subfamily of transcriptional regulators [1–3]. Together with SOX1 and SOX2 it is expressed in neural progenitors where they counteract the activity of proneural proteins and maintain undifferentiated state of progenitor cells [4]. *SOX2* gene, the closest relative of *SOX3*, is one of the core pluripotency factors involved in the regulation of stemness and differentiation [3,5,6]. SOX3 is recognized as one of the earliest neural markers in vertebrates; up to date the role of *Sox3* in neural development has been the most studied aspect of the *Sox3* action.

It was shown that in murine telencephalon *Sox3* is expressed in neural stem/progenitor cells (NP cells) during embryonic development and it is downregulated during neuronal differentiation [7]. In adult mice telencephalon, *Sox3* expression is maintained only in progenitor cells of the adult neurogenic regions, subventricular and subgranular zones [7]. In contrast, during hypothalamic neurogenesis *Sox3* expression is not restricted to neural progenitors, but to developing neurons and is maintained in a subset of differentiated hypothalamic cells through adulthood [7]. Consistent with its expression patterns, *Sox3* plays important roles in the process of neural differentiation, as confirmed by genome-wide binding studies that verified its status as one of the earliest markers of vertebrate neurogenesis. It has been demonstrated that in mouse ES-derived NP cells Sox3 target genes have regulatory roles during development of the CNS [1]. While Sox3 mainly activates genes expressed in NP cells, it also binds to neuronal genes, preventing premature Sox11 binding and their consequent activation [1]. Recent studies have identified Sox3 target sites in murine NP cells in putative enhancers of neurodevelopmental genes, located primarily within the intergenic regions [8]. Furthermore, Sox3 acts as a pioneer factor whose binding to target enhancers establishes local epigenetic changes [1]. Due to functional redundancy between *SoxB1* genes the expression of most NP genes is not affected in *Sox3* null NP cells. Nevertheless, direct Sox3 targets have been identified with expression not rescued by other SoxB1 members [9].

Besides the prominent roles in the process of neural differentiation, there is evidence pointing at *SOX3* as one of the players in the maintenance of human embryonal stem cells (hESCs) identity. Together with SOX2, SOX3 is implicated in the regulation of self-renewal and pluripotency of hESCs [10]. *SOX3* is upregulated after the knockdown of *SOX2* in hESC, keeping the cells in an undifferentiated state, while the self-renewal ability is reduced under these conditions [10]. Moreover, it was established that *Sox1* and *Sox3* can replace *Sox2* during the process of iPSCs (induced pluripotent stem cells) generation from mouse embryonic fibroblasts (mEFs) [11]. Taken together, these data highlight the role of *Sox3* in the selection and proper execution of developmental programs established through complex coordination between *Sox3* and other *SoxB1* genes and their partners.

Reports concerning the mechanisms of *SOX3* regulation during neural differentiation are limited and mainly focused on the transcriptional control of human *SOX3* expression [1,12–17]. In recent years, it was revealed that regulation of developmental genes with dynamic expression patterns is not driven only by transcription factor networks, but also by the epigenome (reviewed in [18,19]). Epigenetic regulation of gene expression is achieved through genomic DNA methylation, post-translational modifications (PTMs) of histones, chromatin remodeling and non-coding RNAs [19]. The complex interplay between these mechanisms represents a mode in which genotype controls phenotype without changes in the DNA sequence. Special efforts are made in an attempt to delineate epigenetic processes underlining the formation of neurons, with an aim to improve stem cell based therapies in neurodegenerative diseases, and to control commitment of pluripotent cells [20]. Epigenetic profiles of pluripotency-associated genes, such as *Oct4*, *Sox2* and *Nanog* have been investigated in several studies, and correlated with dynamic expression of these genes during development [21–23] while epigenetic control of *SOX3* expression remained to great extent understudied.

In the present study we have analyzed epigenetic modifications of the promoters of *SOXB1* genes during early phases of retinoic acid (RA) induced neural differentiation of human embryonal carcinoma NTera2/D1 (NT2/D1) cells with a special focus on *SOX3* gene. We demonstrate that the human *SOX3* gene promoter is extremely hypomethylated both in undifferentiated and RA-induced NT2/D1 cells and that it does not react to treatment with the demethylating agent 5-azacytidine (5-azaC). Furthermore, by employing chromatin immunoprecipitation (ChIP), we show that different regions of *SOX3* gene are enriched in distinct histone PTMs switching throughout the course of RA-induced neural differentiation. The profiles of histone modifications on the *SOX3* promoter differ from those on the *SOX1* and *SOX2* promoters, implying that *SOXB1* genes are controlled by different epigenetic mechanisms.

## Materials and methods

### Cell culture and treatments

NT2/D1 cells, kindly provided by Prof. P.W. Andrews (University of Sheffield, UK) were maintained in Dulbecco's Modified Eagle's medium (DMEM) supplemented with 10% fetal bovine serum (FBS) 4500 mg/L glucose, 2 mmol/L L-glutamine and penicillin/streptomycin (all from Invitrogen^™^, NY, USA), at 37°C in 10% CO_2_ as previously described [24]. Cells were induced to differentiate by addition of 10μmol/L all-trans retinoic acid (Sigma-Aldrich, MO, USA) into the culture media and grown for 2, 4 and 7 days as previously described [24]. For 5-azacytidine treatment (5-azaC, Acros Organics, Belgium), cells were grown for 24h in 1 μmol/L 5-azaC.

### Western blot

Western blot analyses of SOX3, SOX1 and SOX2 expression during RA induction were performed on whole cell lysates (WCL) extracted from uninduced NT2/D1 cells and cells induced with RA for 2, 4 and 7 days. For the analyses of SOX3 expression during treatments with 5-azaC WCL were isolated from NT2/D1 cells and cells treated with 5-azaC. WCL were isolated and western blots were performed as previously described [15] using anti-SOX3 (Abcam, ab42471), anti-SOX1 (Abcam, ab109290), anti-SOX2 (Active Motif, 39823), anti-α-tubulin (Calbiochem, DM1A) and anti- GAPDH (Abcam, ab9484), anti-caspase-3 (Cell Signaling, 9662), followed by the incubation with horseradish peroxidase-conjugated anti-mouse and anti-rabbit IgG secondary antibodies (Amersham Biosciences, NJ, USA, diluted 1:10000). Immunoreactive bands were detected by Immobilion Western Chemiluminescence substrate HRP (Millipore, MA, USA). Density of protein bands on blots were quantified using ImageJ software (https://imagej.nih.gov/). Data from at least 3 independent experiments were normalized by the amount of α-tubulin and presented relative to the corresponding value for untreated cells.

### Real time PCR analysis

Total RNA was isolated using TRI-Reagent (Ambion, Invitrogen,USA) according to the manufacturer's instructions. RNA was treated with DNaseI using a DNA-Free^™^ kit (Ambion, Invitrogen, USA) and subjected to cDNA synthesis. Total RNA (1 μg) was reverse transcribed using High Capacity cDNA Reverse Transcription Kit (Applied Biosystems, USA) according to the manufacturer's protocol. cDNAs were subjected to real time PCR using Power SYBR Green PCR Master Mix (Applied Biosystems, USA) in 7500 Real Time PCR Systems (Applied Biosystems, USA). Following primers were used:

*SOX3*
     5’- GGGGAGGGCTGAAAGTTTTG-3’ (forward)
     5’- ACACAGCGATTCCCAGCCTA-3’ (reverse)
*Nanog*
     5’- GGTCCCGGTCAAGAAACAGA -3’ (forward)
     5’- TCTGGAACCAGGTCTTCACC -3’ (reverse)
*Dbx1*
     *5’- AAGCTGGGCCTGAAAGACTC -3’ (forward)*
     *5’- CCTCCTCCTCCTCTTCGTTC -3’ (reverse)*
*SOX2*
     5’- CCCCTGGCATGGCTCTTGGC -3’ (forward)
     5’- TCGGCGCCGGGGAGATACAT -3’ (reverse)
*GAPDH*
     *5’- G*GACCTGACCTGCCGTCTAG *-3’* (forward)
     *5’-* CCACCACCCTGTTGCTGTAG *-3’* (reverse)

All samples were measured in triplicate and the mean value was considered. The relative levels of *SOX3*, *SOX2* and *Nanog* expression were determined using a comparative quantification algorithm where the resulting ΔΔCt value was incorporated to determine the fold difference in expression (2^−ΔΔCt^). Relative *SOX3*, *SOX2* and *Nanog* mRNA levels were presented as a fold change in gene expression normalized to *GAPDH* and relative to the value in untreated NT2/D1 cells, which was set as 1. The expression level of *Dbx1* was analyzed as 2^−ΔCt^ due to the low levels of *Dbx1* mRNA in undifferentiated NT2/D1 cells.

### Immunostaining

NT2/D1 cells were plated on coverslips and cultured in the absence (for 2 days) or presence (for 2, 4 and 7 days) of RA. Following fixation in 4% paraformaldehyde for 20 min at room temperature and permeabilization in 0.1% Triton X 100, cells were incubated in blocking solution, 10% normal goat serum and 1% bovine serum albumin (BSA) in PBS for 1 h at room temperature. Primary antibodies diluted in 1% BSA (Sigma Aldrich, USA), 0.1% Triton X 100 in PBS were applied overnight at 4°C as follows: mouse monoclonal anti OCT-3/4 (sc-5279, diluted 1: 100; Santa Cruz Biotechnology, Inc) and rabbit polyclonal anti SOX3 (sc-20089, diluted 1: 100; Santa Cruz Biotechnology, Inc). Coverslips were washed three times for 10 min in 0.1% Triton X 100 in PBS and incubated with biotinylated goat anti rabbit IgG (1: 500; Vector, USA) for 1 h at room temperature in 1% BSA, 0.1% Triton X 100 in PBS followed by DyLight 488^®^ streptavidin (1: 1000; Vector Laboratories, USA) and Alexa FluorH 594, (1: 500; InvitrogenTM) diluted in PBS for 1 h at room temperature. Nuclei were stained with 0.1 mg/ml 4′,6 diamino phenylindole (DAPI; Sigma Aldrich). Samples were viewed and images were taken using a Leica TCS SP8 confocal microscope and Leica Microsystems LAS AF TCS SP8 software (Leica Microsystems, Germany).

### RT-PCR analysis

Total RNA was isolated, subjected to DNaseI treatment and reversely transcribed as described in previous subsection. The synthetised cDNAs were used as templates for PCR amplifications with primers specific for *SOX1* and *GAPDH* (as in previous subsection) as a loading control. Primers for *SOX1* amplification were as follows:

5’- GCACCACTACGACTTAGTCCG -3’ (forward)
5’- AGACCTAGATGCCAACAATTGG -3’ (reverse)

RT-PCRs were performed in 20 ul reactions using KAPA 2G Fast HotStart Ready Mix (Kapa Biosystems) according to manufacturer’s protocol. Obtained products were separated electrophoretically on 2% agarose gel and visualized using ethidium bromide staining. Quantification of obtained bands was performed using ImageJ software (https://imagej.nih.gov/ij/).

### Methylation-specific PCR

For MSP analyses, direct sodium-bisulfite conversion of uninduced NT2/D1 cells and cells induced with RA for 2, 4 and 7 days was performed using EZ DNA Methylation-Direct^™^ Kit (Zymo Research Corporation, CA, USA). For each conversion 10^5^ cells were used and protocol provided by the manufacturer strictly followed. Upon conversion the reaction recovery rate was considered as 100% and hence concentration of DNA was not measured. Converted DNA samples were used as templates for PCR amplification using KAPA 2G Fast HotStart Ready Mix (Kapa Biosystems) and cycling conditions 95°C for 5 minutes; 95°C for 30 seconds, 60°C for 20 seconds, 72°C for 2 minutes, for 35 cycles. MSP primers were designed using MethPrimer web-based tool [25]. Following primers were used for amplification of methylated (M) versus unmethylated (U) *SOX3* gene promoter sequence:

M:
     5'- GTAGATTGTGAATGCGATTTGTTC -3'
     5'- GATAAAAAAACCCTAAAACTCCGTC -3'
U:
     5'- GGTAGATTGTGAATGTGATTTGTTT -3'
     5'- ACAATAAAAAAACCCTAAAACTCCAT -3'

For MSP analysis of *SOX1* gene promoter following primers were used for amplification of methylated (M) versus unmethylated (U) sequence:

M:
     5'- AATTTTTTATTTGCGAGTCGAATC -3'
     5'- AAAAACCTAAAACATAAACGACCG -3'
U:
     5'- GAAATTTTTTATTTGTGAGTTGAATTG -3'
     5'- AAAACCTAAAACATAAACAACCAAA -3'

Obtained products were separated electrophoretically on 2% agarose gel and visualized using ethidium bromide staining.

### Extraction and sodium-bisulfite conversion of genomic DNA

Pellets collected from uninduced NT2/D1 cells and cells induced with RA for 2, 4 and 7 days were resuspended in TSM buffer [140 mM NaCl, 10 mM Tris (pH 7.4), 1.5 mM MgCl2, 0.5% NP40]. After short centrifugation, pellets were lysed in nuclei dropping buffer (75 mM NaCl, 24 mM EDTA, 0.2mg/ml proteinase K, 0.5% SDS). High molecular weight DNA was extracted using phenol-chlorophorm-isoamylalcohol extraction, and precipitated with sodium acetate and isopropanol.

Sodium-bisulfite conversion of isolated genomic DNA was performed using EZ DNA Methylation-Lightning^™^ Kit (Zymo Research Corporation, CA, USA). For each conversion 2 μg of DNA was used and protocol provided by the manufacturer strictly followed. Upon conversion the reaction recovery rate was considered as 100% and hence concentration of DNA was not measured.

### PCR amplification of bisulfite converted *SOX3* promoter

PCRs were performed in 60 μl reactions using KAPA 2G Fast HotStart Ready Mix (Kapa Biosystems) according to the manufacturer's protocol. Bisulfite converted DNA from uninduced NT2/D1 cells and cells induced for 2, 4 and 7 days with RA were used as a template to amplify 2nd CpG island within *SOX3* promoter. PCR cycling conditions were 95°C for 10 minutes; 95°C for 20 seconds, 50–55°C for 20 seconds, 60°C for 2 minutes, for 40 cycles. PCR reactions were performed using non-modified forward primer and 5’-biotin-labeled reverse primer listed below. BSP primers were designed using MethPrimer tool [25]. Indicated primers positions were determined relative to TSS.

2^nd^ CpG island:
     5’-AAGGGGTTTAGTTAGAGTTTA-3’ (-6 to +15)
     5’-AATCTCCAAAAAACTATACAT-3’ (+253 to 273)

### Pyrosequencing

Pyrosequencing of amplified biotinylated PCR products was performed by commercial service at Queen Mary University of London, Genome Centre, London on Pyromark MD system, Biotage (Qiagen Pyrosequencing for 2^nd^ CpG island was performed with primers:

SEQ3
     5’-TTTAGGTAGATTGTGAATG-3’
SEQ4
     5’-TTGGTTTATAGGTTTTTAAG-3’

In the obtained pyrograms, the amount of C relative to the sum of the amounts of C and T at each CpG site is calculated as percentage of methylation level of indicated CpG site. Total methylation levels of 2^nd^ CpG island within *SOX3* promoter were calculated as average values of every individual CpG sites methylation levels.

### Chromatin immunoprecipitation

Untreated NT2/D1 cells and NT2/D1 cells treated with 10 μM RA for 2, 4 and 7 days were crosslinked with 1% formaldehyde in DMEM for 10 minutes at room temperature and rinsed with cold PBS. Crosslinking reaction was stopped with 0.125 mM glycine in cold PBS for 5 minutes. Cells were lysed with lysis buffer [5 mM Pipes (pH 8.0), 85 mM KCl, 0.5% NP-40, Protease inhibitor cocktail]. Cells were dounced on ice and nuclei separated by centrifugation. Nuclei were lysed in sonication buffer (50 mM Tris pH 8.0, 10 mM EDTA, 0.1% SDS, 0.5% deoxycholic acid, Protease inhibitor cocktail (Roche Diagnostics GmbH, Switzerland)]. Chromatin was sonicated to fragments of 500–1500 bp. Precleared chromatin in immunoprecipitation buffer [50 mM Tris (pH 8.0), 10 mM EDTA, 0.1% SDS, 0.5% deoxycholic acid, 150 mM LiCl, Protease inhibitor cocktail (Roche Diagnostics GmbH)] was incubated overnight with following antibodies: anti-Flag (Sigma Aldrich, F3165), anti-H3 (Abcam, 1791), anti-H3K4me3 (Active Motif, 39159), anti-H3K79me2 (Abcam, 3594), anti-H2B (Abcam, 1790), anti-H2BK16ac (Active Motif, 39121), anti-H2BK120ac (Active Motif, 39119), anti-H2BK5ac (Active Motif, 39123). Following day, chromatin and antibodies were incubated for 4 hours with Protein G agarose (KPL, USA) saturated with salmon sperm DNA and BSA overnight. Samples were centrifugated and Flag supernantants saved for Inputs. Resins were washed 5 times with RIPA buffer [10 mM Tris (pH 8.0), 1 mM EDTA, 0.5 mM EGTA, 0.1% SDS, 0.1% deoxycholic acid, 140 mM NaCl, 1% Triton X-100, Protease inhibitor cocktail(Roche Diagnostics GmbH, USA), 1 mM PMSF], followed by wash with LiCl buffer [0.25 M LiCL, 0.5% NP-40, 0.5% deoxycholic acid, 10 mM Tris (pH 8.0), 1 mM EDTA] and wash in TE buffer, pH 8.0. Resins were resuspended in TE buffer, and cross-links reversed by overnight incubation with RNase A (Sigma Aldrich, MO, USA) at 65°C. Following day all samples were adjusted to 0.5% SDS and treated with 20 μg of Proteinase K (Sigma Aldrich, MO, USA) for immunoprecipitated samples and 40 μg for Input samples for 3 hours at 50°C. DNA was extracted with phenol/chlorophorm/isoamylalcohol and precipitated overnight at -20°C with 3M sodium-acetate, tRNA (Sigma Aldrich, MO, USA) and ethanol. Following day DNA samples were centrifuged and DNA resuspended in H_2_O.

DNA sequences of *SOX3* (RefSeq NM_005634) upstream (-673/-578), *SOX3* core promoter (-224/-63), *SOX3* 5’ downstream coding region (+503/+606), *SOX2* (RefSeq NM_003106) promoter (-107/+56) and *SOX1* (RefSeq NM_005986) promoter (-147/+25) were analyzed using qPCR with following primers:

*SOX3* upstream
     5’–GCAGTCCTGAAGCCTGTCTC-3’ (forward)
     5’–GCGTCTCCAAGAAGCTCTCC-3’ (reverse)
*SOX3* core promoter
     5’- AGGGCTCCCCGAACTTTT-3’ (forward)
     5’- GCTGGGCCCCTTATATACCT-3’ (reverse)
*SOX3* downstream
     5’- TGGAGAACCCCAAGATGCAC-3’ (forward)
     5’- CTTGGCCTCGTCGATGAATG-3’ (reverse)
*SOX2* promoter
     5’- GCCCCCTTTCATGCAAAACC-3’ (forward)
     5’- CTCTGCCTTGACAACTCCTG-3’ (reverse)
*SOX1* promoter
     5’- ACCCCTCCCCATTCTTCTCT-3’ (forward)
     5’- CAGGTCGGTCTCCATCATCA-3’ (reverse)

The enrichment was calculated relative to Flag and normalized against H3 or H2B. In comparative experiments, the enrichment in undifferentiated cells was assigned the value 1 and other samples were normalized to this value [26]. Results are representative of duplicated qPCR reactions from three ChIP experiments (biological replicates).

### Statistics

Data were presented as mean ± S.D. and were analyzed using Student’s T test. *P<0.05 was considered statistically significant.

## Results

### Analysis of human *SOX3* gene expression during early phases of neural differentiation of NT2/D1 cells

**Fig 1:**
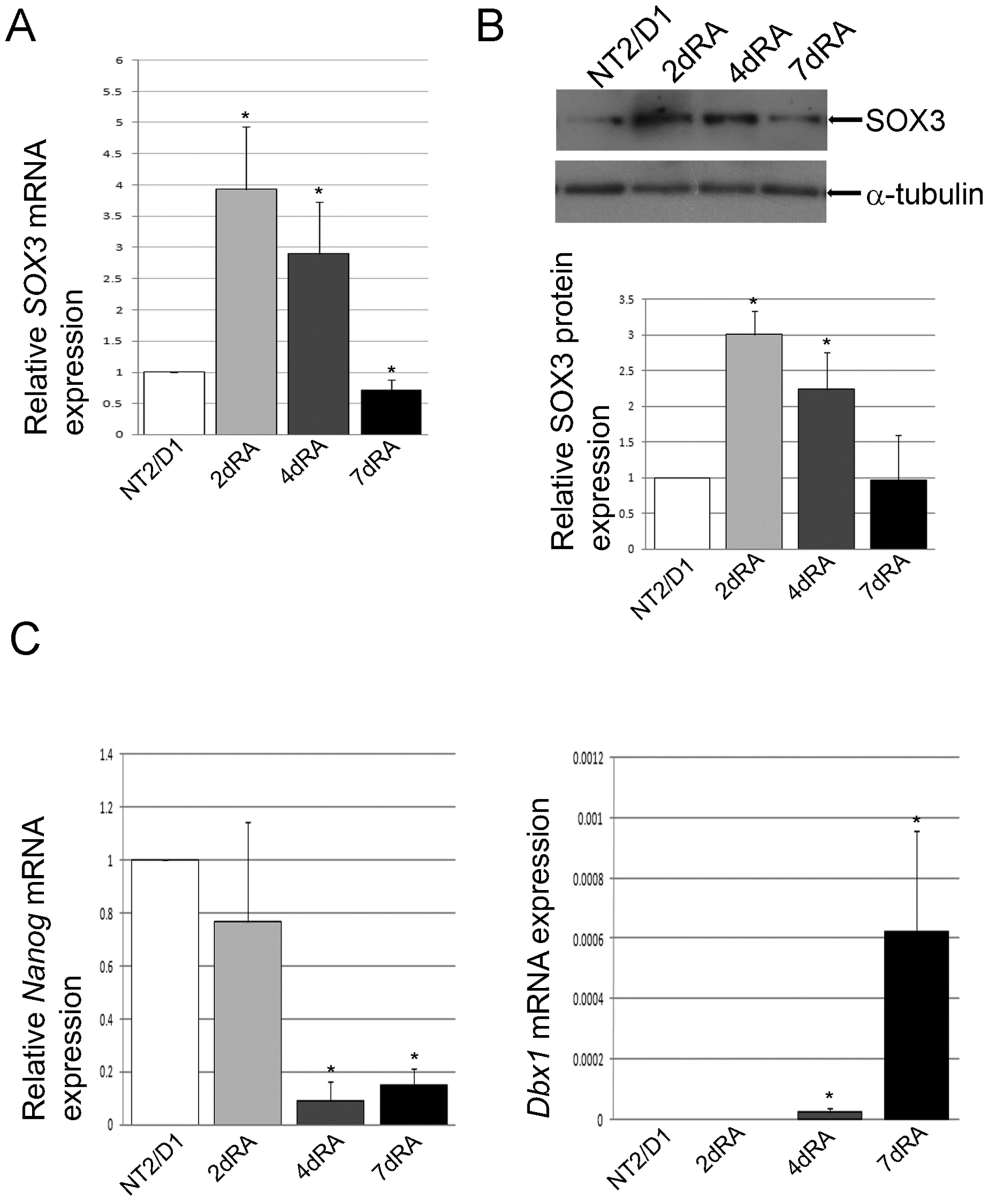
*SOX3* is upregulated during early phases of RA induced neural differentiation of NT2/D1 cells. (A) Real-time PCR analysis of *SOX3* expression in untreated and RA treated NT2/D1 cells (2, 4 and 7 days). Data were normalized by the amount of *GAPDH* mRNA and presented relative to the corresponding value for untreated cells, and are means ± S.D.,*P<0.05 from triplicate data. (B) Western blot analysis of SOX3 protein in whole cell lysates of untreated and NT2/D1 cells treated with RA for 2, 4 and 7 days. SOX3 protein quantities were expressed relative to untreated NT2/D1 cells (set at 1) and presented as mean ± S.D. of at least three independent experiments; *P<0.05. α-tubulin was used as loading control. Representative blots are shown. (C) Real-time PCR analyses of pluripotency marker *Nanog* and neural marker *Dbx1* expression patterns during early phases of RA induced neural differentiation of NT2/D1 cells. *Nanog* expression data were normalized by the amount of *GAPDH* mRNA and presented relative to the corresponding value for untreated cells, and are means ± S.D., *P<0.05 from triplicate data. *Dbx1* expression levels were normalized by the amount of *GAPDH* mRNA and calculated as 2^-Δct^. Data are presented as means ± S.D., *P<0.05 from triplicate data.

SOX3 is considered as one of the key regulators of neural development in vertebrates [27]. We analyzed its expression in the early stage of RA-induced neural differentiation of NT2/D1 cells. Due to their similarity with hESC and the property to differentiate into morphologically and physiologically mature neurons after exposure to RA, NT2/D1 cells represent an appropriate *in vitro* model to study the process of human neural differentiation [24,28,29]. Although derived from human teratocarcinoma, these cells display properties of neural progenitor cells [30]. Following induction with RA, NT2/D1 cells loose expression of neuroepithelial markers and acquire expression of neuronal markers, yielding NT2N neurons that maintain a stable neuronal phenotype, form functional synapses, do not divide, and therefore have been used in various studies as an alternative graft sources in transplantation therapy for ischemia [30,31]. Moreover, in our previous studies and reports made by other groups, NT2/D1 cell line was used for the analyses of human *SOX* genes expression and regulation [12–15,17,23,32–37]. We have previously shown that the treatment of NT2/D1 cells with RA triggered an early (48h) increase in the expression of *SOX3* gene [35]. In the present study, we have expanded this analysis by following SOX3 expression in longer time points (4 and 7 days). By qRT-PCR and Western blot, we demonstrate significant upregulation of SOX3 after exposure of NT2/D1 cells to RA, at the mRNA (Fig 1A) and protein levels (Fig 1B). SOX3 shows a peak at 2 days of RA treatment, followed by decrease in protein and mRNA levels (Fig 1A and 1B).

To verify pluripotency of NT2/D1 cells and confirm the differentiation status after RA induction, we analyzed the expression of Nanog, one of the core pluripotency factors [38]. We also analyzed the expression of Dbx1, a homeodomain protein with a critical role in the establishment of V0 and V1 interneurons [39] and a direct target of Sox3 in neural precursors [9]. We observed a significant decrease in *Nanog* mRNA levels 4 days following RA treatment, confirming the exit of NT2/D1 cells from the pluripotency (Fig 1C). At day 4, the cells show upregulated expression levels of *Dbx1* (Fig 1C). Thus, the expression patterns of SOX3, *Nanog* and *Dbx1* confirm the onset of neural fate commitment.

**Fig 2:**
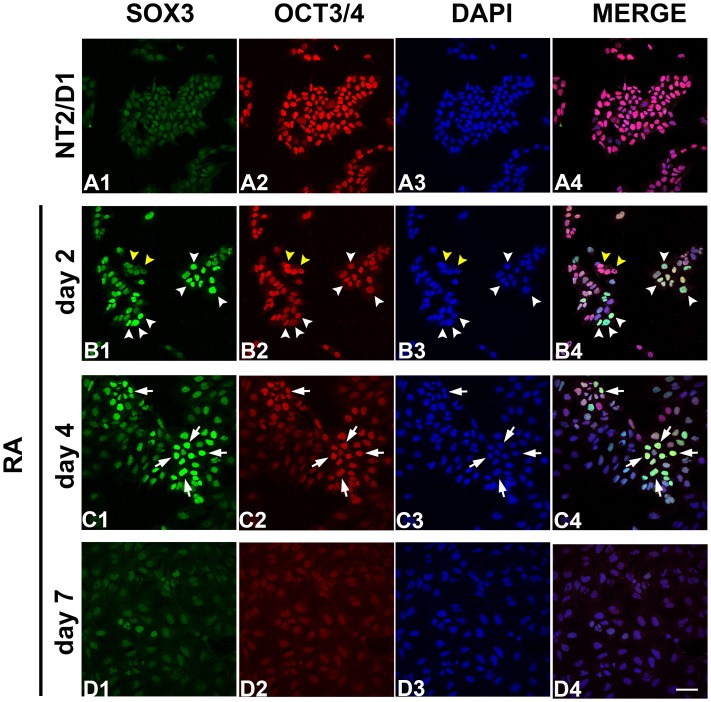
Immunocytochemical co-localization of SOX3 and OCT4 during early phases of RA-induced neural differentiation of NT2/D1 cells. Immunocytochemical detection of SOX3 and OCT4 in untreated NT2/D1 cells (A1-A4) and NT2/D1 cells treated with RA for 2 (B1-B4), 4 (C1-C4), and 7 (D1-D4) days. Cells with high level of SOX3/low level of OCT4 expression are designated by white arrowheads in panels B1-B4. Cells with low level of SOX3/high level of OCT4 expression are designated by yellow arrowheads in panels B1-B4. Cells that are highly imunopositive for both markers are designated by white arrows in panels C1-C4. Cell nuclei were stained with DAPI (A3, B3, C3, D3). Scale bar: 50 μm.

In order to further examine the temporal pattern of SOX3 protein expression during early phases of neural differentiation in our model system, we analyzed its co-expression with stemness marker OCT4 by immunocytochemistry. We have previously demonstrated that OCT4, together with Nanog, is downregulated during the first week of neural differentiation of NT2/D1 cells [34]. Herein, we confirmed that strong OCT4 immunoreactivity detected in undifferentiated NT2/D1 cells (Fig 2A2 and 2A4) declined in most of the cells following 2 (Fig 2B2 and 2B4) and 4 days (Fig 2C2 and 2C4), but almost completely disappeared after 7 days of RA induction (Fig 2D2 and 2D4). As shown in Fig 2, we detected a low level of SOX3 protein expression in NT2/D1 cells prior to the differentiation (Fig 2A1 and 2A4) that sharply increased after 2 days of RA treatment. Induction of SOX3 in most of the cells coincided with reduced level of OCT4 protein expression suggesting that these two transcription factors are oppositely regulated at initial periods of neural differentiation (Fig 2, arrowheads in B1, B2, B3, B4). However, as differentiation proceeded, only populations of cells which retained medium OCT4 protein expression level were also immunoreactive for SOX3 (Fig 2, arrows in C1, C2, C3, C4). Finally, at the end of the differentiation protocol, at day 7, expression level of SOX3 was barely detectable (Fig 2D1 and 2D4). Taken together, our results demonstrated that SOX3 protein is transiently expressed in specific developmental stage of NT2/D1 cells during initiating phases of *in vitro* neural differentiation. This prompted us to further investigate possible epigenetic mechanisms implicated in the regulation of SOX3 expression.

### *In silico* analysis of human *SOX3* promoter

**Fig 3:**
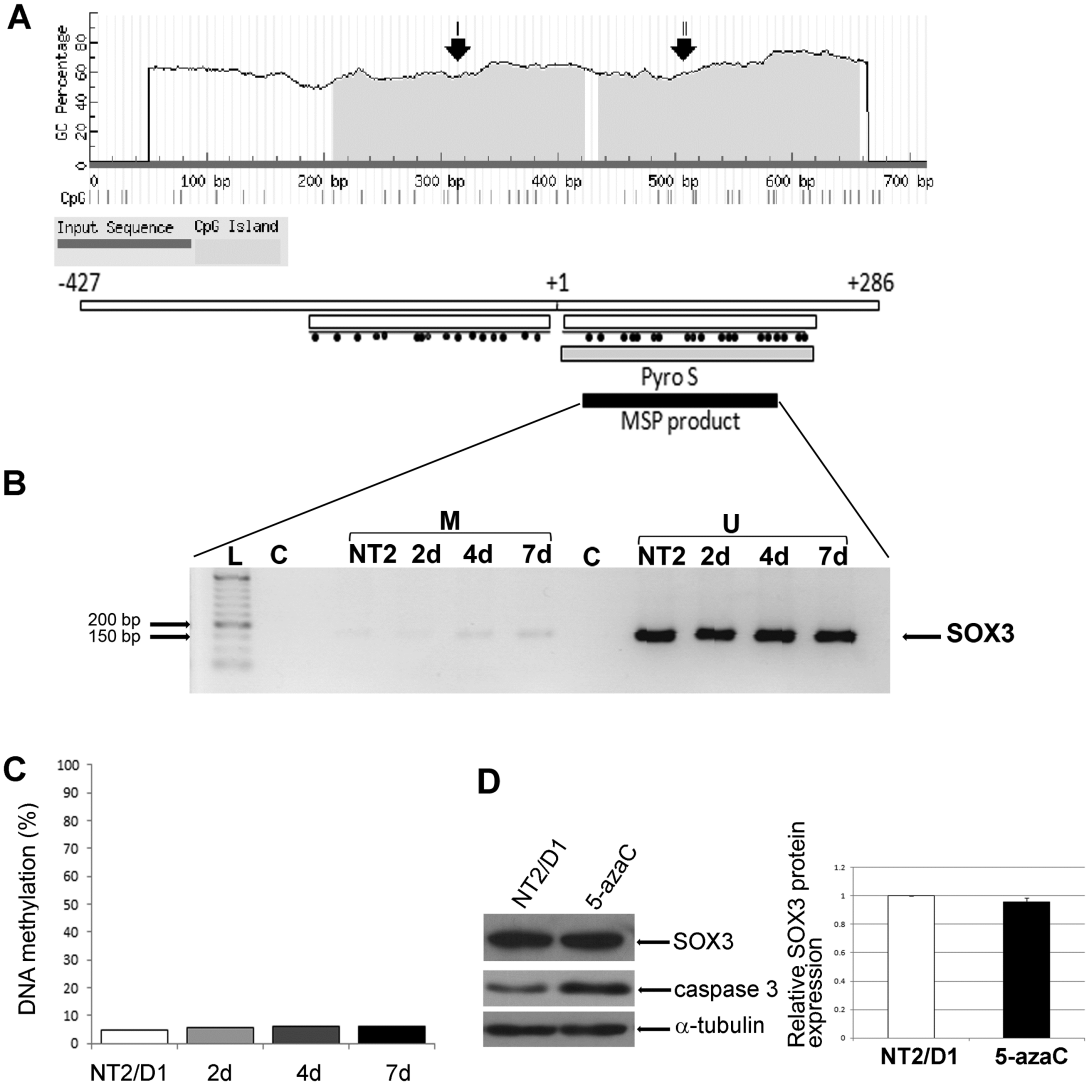
Human *SOX3* promoter is hypomethylated during early phases of neural differentiation of NT2/D1 cells. (A) CpG islands localization within the human *SOX3* promoter determined by MethPrimer online software. Arrows I and II indicate CpG island regions. Bellow MethPrimer graph is a schematic representation of the human *SOX3* promoter and regions analyzed using MSP and pyrosequencing (Pyro S). Individual CpGs are represented as black dots. Numbers represent end points of the analyzed promoter region relative to tss (+1). (B) Analysis of *SOX3* promoter methylation in untreated (NT2/D1) and cells treated with RA in indicated time points (2, 4 and 7 days) by MSP. Product obtained with primers corresponding to methylated (M) 2^nd^ CpG island within *SOX3* promoter and product obtained with primers corresponding to unmethylated (U) 2^nd^ CpG island within *SOX3* promoter were separated on agarose gel. (C) Quantitative analysis of *SOX3* promoter methylation in untreated (NT2/D1) and cells treated with RA in indicated time points (2, 4 and 7 days) by pyrosequencing of the 2^nd^ CpG island. Bars indicate mean levels of methylation of *SOX3* promoter in each time point. (D) Effects of 1μM 5-azaC treatment on the expression of SOX3 protein in NT2/D1 cells. Quantity of SOX3 protein in treated cells was calculated relative to untreated NT2/D1 cells (set at 1) and presented as the means ± S.D. of at least three independent experiments; *P<0.05. Caspase-3 expression was used as a positive control. Representative blots are shown.

Functionally, the promoter of human *SOX3* gene is 713 bp in length [12] and we analyzed it with the MethPrimer tool [25]. The analysis was conducted using default criteria for CpG island prediction, and it has revealed the presence of 2 CpG islands within the human *SOX3* promoter (Fig 3A). The island with 17 CpGs dinucleotides (from 209–423 bps) corresponds approximately to the minimal *SOX3* promoter [12], while the second CpG island with 22 CpGs (434–657 bps) encompasses the *SOX3* TSS ending downstream of the second ATG codon (Fig 3A) [12].

### The methylation profile of *SOX3* promoter during early phases of neural differentiation

We sought to determine potential dynamic changes in the methylation levels of these CpG islands during the early phases of neural differentiation of NT2/D1 cells. First, we have employed methylation-specific PCR (MSP) to assess methylation status of the human *SOX3* promoter in undifferentiated NT2/D1 cells and cells treated with RA for 2, 4 and 7. While the 1^st^ CpG island could not be subjected to MSP analysis due to the sequence-based difficulties in MSP primers design, 2^nd^ CpG island was successfully amplified. As shown in Fig 3B, products obtained with primer set corresponding to unmethylated DNA were highly abundant, in comparison with products obtained with primer set corresponding to methylated DNA. These results suggested that second CpG island within the *SOX3* promoter is unmethylated in undifferentiated NT2/D1 cells and that this hypomethylation is sustained during RA-induction of these cells. In order to elucidate these findings in more detail, we employed bisulfite pyrosequencing.

We found 1^st^ CpG island to be difficult for the analysis due to GC-rich sequence which could give rise to homopolymers and secondary structures following bisulfite conversion of DNA. These structures prevent optimal amplification and subsequent pyrosequencing of the region encompassing 1^st^ CpG island within the *SOX3* promoter and therefore we proceeded with the analysis of the second island. Genomic DNA isolated from undifferentiated NT2/D1 cells and cells treated with RA for 2, 4 and 7 days were subjected to the sodium-bisulfite conversion. Converted DNA was used as a template to amplify 2^nd^ CpG island within *SOX3* promoter using non-modified forward primer and biotin-labeled reverse primer and PCR products were analyzed by pyrosequencing [40]. We demonstrated hypomethylation of the *SOX3* promoter in undifferentiated NT2/D1 cells (Fig 3C), which is preserved during RA-induced neural differentiation (Fig 3C). Average methylation of *SOX3* does not exceed 10% at all timepoints analyzed, as shown in Fig 3C. The data suggest that methylation of *SOX3* does not correlate with the dynamic changes of *SOX3* expression during the initial phases of neural differentiation.

In order to confirm these data, we treated NT2/D1 cells with 5-azaC, a demethylating agent acting during DNA replication and cell division [41,42] followed by the analysis of the SOX3 expression levels (Fig 3D). Endogenous caspase-3, previously shown to be upregulated following treatment of NT2/D1 cells with nucleoside drugs [43], was used as a positive control (Fig 3D). As expected, treatment with 5-azaC did not induce any significant change in SOX3 protein levels (Fig 3D). The lack of upregulation of the SOX3 protein expression upon exposure to 5-azaC further supports the idea that methylation is not a mechanism governing RA-induced activation of *SOX3* expression in NT2/D1 cells.

### Histone modifications profiles on *SOX3* gene during early phases of RA-induced neural differentiation of NT2/D1 cells

**Fig 4:**
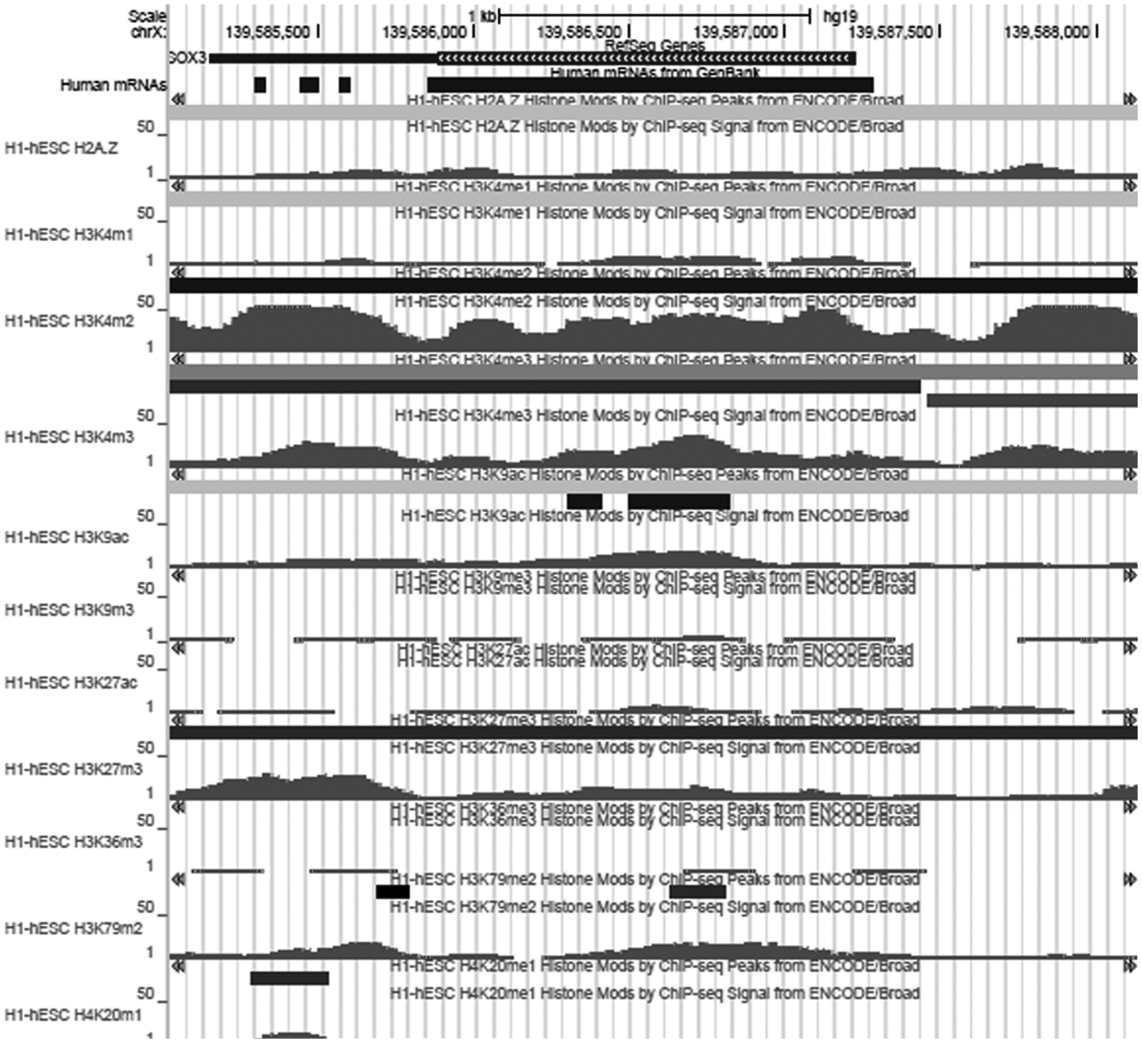
UCSC genome browser tracks showing histone modifications around *SOX3* locus in H1-hESCs. Shown from top to bottom is layered H2A.Z mark; enhancer- and promoter-associated H3K4me1, H3K4me2 and H3K4me3 marks; active marks H3K9ac, H3K9me3 and H3K27ac; repressive mark H3K27me3; marks associated with coding regions H3K36me3, H3K79me2 and H4K20me1. Arrows in the first track depict direction of transcription.

Numerous reports highlighted the importance of histone covalent PTMs in the control of nucleosome dynamics during the process of differentiation induced by the environmental stimuli [44]. In order to check the status of histone modifications in the *SOX3* gene in pluripotent cells, we examined ChIP-Seq reads in human ES cell line H1 (H1-hESC) using ENCODE datasets (Fig 4) (GRCh37/hg19; http://genome.ucsc.edu). ChIP-Seq signals for H3K4me2 and H3K4me3 in the promoter and the *SOX3* coding region are high (Fig 4). Other markers are absent from the *SOX3* promoter (H3K9ac and H3K27ac) and coding region (H3K36me3 and H4K20me1), and H3K79m2 is low within the coding region (Fig 4). Furthermore, H3K27me3 overlaps with the 3’ region, but it is not present at the *SOX3* promoter (Fig 4). These histone signatures indicate permissive promoter with low transcriptional activity in pluripotent cells, consistent with low expression levels of SOX3 in hESCs [10].

**Fig 5:**
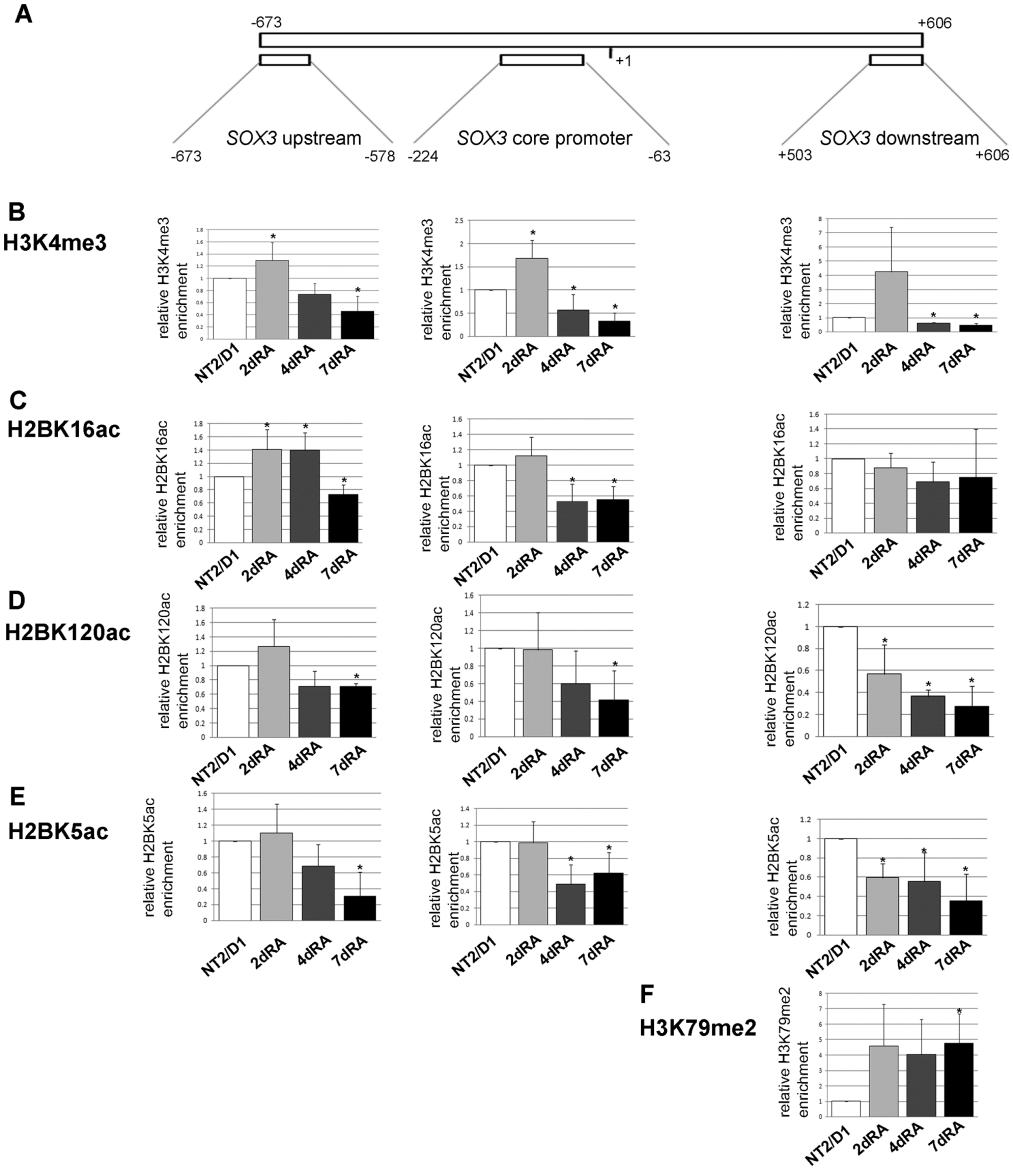
ChIP-qPCR analysis of *SOX3* regulatory regions. (A) Schematic representation of the human *SOX3* gene indicating *SOX3* upstream (left tile), *SOX3* core promoter (center tile) and *SOX3* downstream (right tile) regions analyzed by ChIP. The positions of analyzed regions, relative to TSS, are indicated. (B-F) ChIP-qPCR results for the indicated histone modification that correspond to *SOX3* regions presented above. The enrichment was calculated relative to Flag and normalized against H3 or H2B. In comparative experiments, the enrichment in undifferentiated cells was assigned the value 1 and other samples were normalized to this value. Each ChIP experiment was repeated three times (biological replicates) followed by duplicate qPCR reactions. Results are presented as the mean ± S.D., *P<0.05.

In order to investigate histone modifications on *SOX3* during neural differentiation of NT2/D1, we employed ChIP using a range of specific antibodies, monitoring by qPCR three regions: a region ~600bp upstream of the TSS (SOX3 upstream), the *SOX3* core promoter and a region within *SOX3* gene, ~550bp downstream from TSS (SOX3 downstream) (Fig 5A). We detected enrichment of H3K4me3 on the core promoter region (Fig 5B). Upon RA-induction the level of H3K4me3 increased 1.6 times on the *SOX3* core promoter within the first 2 days of RA induction, followed by a statistically significant drop at days 4 and 7 (Fig 5B). This profile mirrors the expression levels of the *SOX3* gene (Fig 1A and 1B), providing evidence of a link between H3K4me3 and *SOX3* transcription. Moreover, the region upstream of the *SOX3* TSS is undergoing similar, but less prominent changes in H3K4me3 levels during the course of differentiation (Fig 5B).

Next, we investigated profiles of H2B acetylation of lysine residues 5, 16 and 120 (Fig 5C–5E). It has been shown that acetylation of histones neutralizes lysine charges, thus affecting nucleosome stability and promoting DNA accessibility and transcriptional activation [45,46]. We demonstrate a statistically significant decrease in the levels of H2BK16ac, H2BK120ac and H2BK5ac on the core promoter of the *SOX3* gene throughout the course of RA induction of NT2/D1 cells (Fig 5C–5E). Surprisingly, we did not observe an increase in H2B acetylation 2 days after RA introduction, the time point corresponding to the highest *SOX3* promoter activity. We analyzed the region upstream of *SOX3* TSS and observed changes in the H2B acetylation levels (Fig 5C–5E) correlating with the expression profile of *SOX3* (Fig 1A and 1B).

In the *SOX3* coding region, we detected a profile of H3K4me3 similar to the core promoter with more prominent increase on day 2 of RA-induction (Fig 5B). We speculate this increase is a reflection of equivalent changes in this mark on the promoter throughout the course of early differentiation. It has been demonstrated that activated transcription affects the levels of H3K4me3 in coding regions of genes and it is accompanied by the shift in the distribution of this mark on the promoter and coding region [47]. Moreover, the profiles of H2B acetylation in the coding region are similar to the ones in the core promoter, with lower abundance of all three marks at days 4 and 7 of RA-induction (Fig 5C–5E). Finally, in the coding region of *SOX3* gene we analyzed the pattern of H3K79me2, a mark associated with elongating RNA Pol II [48,49], and observed an increase after the induction of neural differentiation (Fig 5F). These findings are consistent with the existing data indicating that this modification is enriched in exons and drives the elongation phase of the transcription [48]. Interestingly, the levels of H3K79me2 remain significantly higher, 4-fold approximately, even at day 7 of RA-induction, a time point with the lowest SOX3 expression.

### Analysis of human *SOX1* gene expression, methylation status and histone modifications profiles during the early phases of neural differentiation of NT2/D1 cells

**Fig 6:**
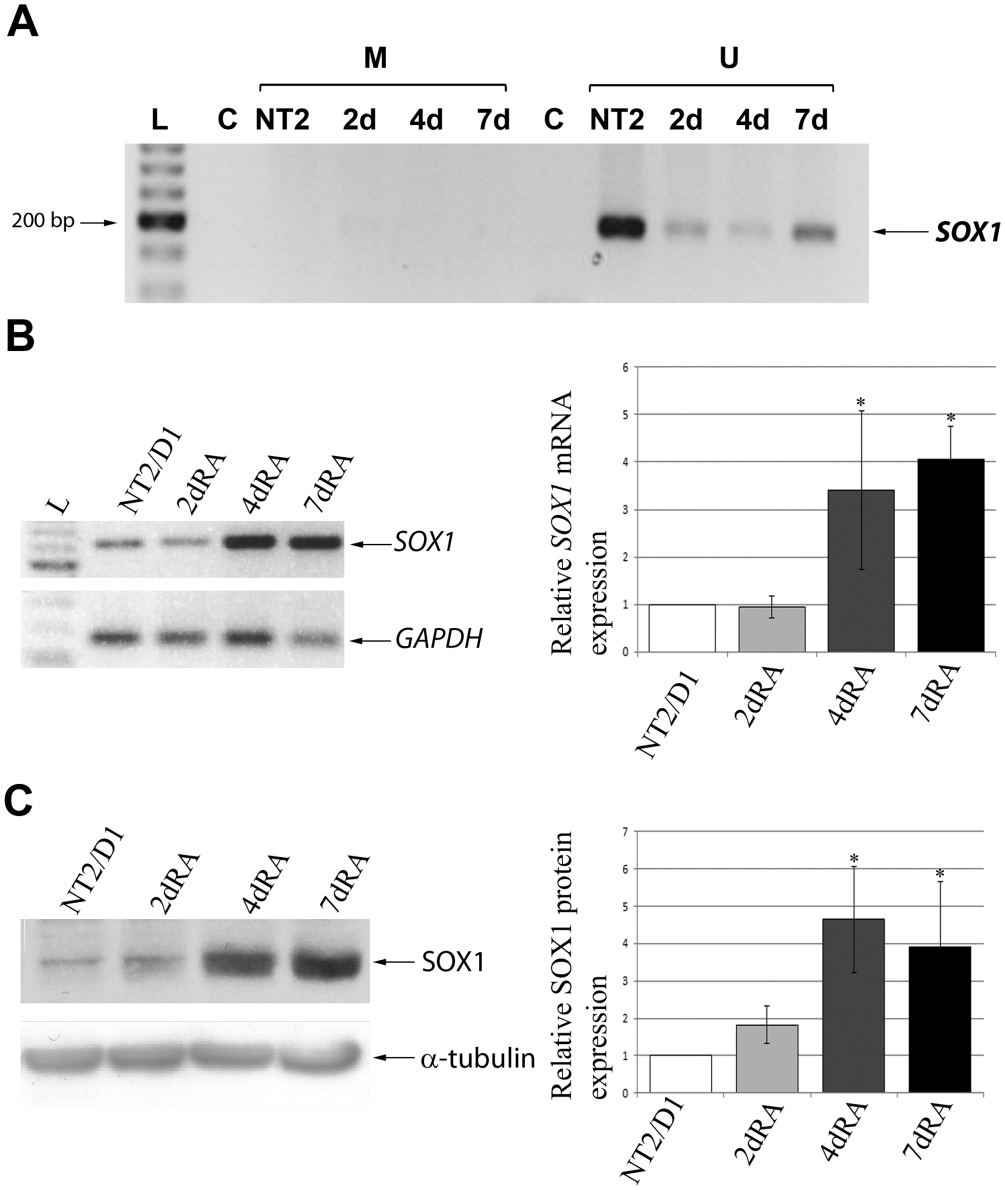
SOX1 is upregulated and hypomethylated during early phases of RA induced neural differentiation of NT2/D1 cells. (A) Analysis of *SOX1* promoter methylation in untreated (NT2/D1) and cells treated with RA in indicated time points (2, 4 and 7 days) by MSP. Product obtained with primers corresponding to methylated (M) *SOX1* promoter and product obtained with primers corresponding to unmethylated (U) *SOX1* promoter were separated on agarose gel. (B) RT-PCR analysis of *SOX1* expression in untreated and RA treated NT2/D1 cells (2, 4 and 7 days). Data were normalized by the amount of *GAPDH* mRNA and presented relative to the corresponding value for untreated cells, and are means ± S.D., *P<0.05 from triplicate data. (C) Western blot analysis of SOX1 protein in whole cell lysates of untreated and NT2/D1 cells treated with RA for 2, 4 and 7 days. SOX1 protein quantities were expressed relative to untreated NT2/D1 cells (set at 1) and presented as mean ± S.D. of at least three independent experiments; *P<0.05. α-tubulin was used as loading control. Representative blots are shown.

We analyzed the expression pattern of *SOX1/*SOX1 mRNA and protein levels in differentiating NT2/D1 cells. As shown in Fig 6, we detected low levels of *SOX1* mRNA (Fig 6B) and SOX1 protein (Fig 6C) in uninduced NT2/D1 cells. At day 4 of RA treatment significant increase in *SOX1*/SOX1 mRNA and protein levels was observed and remained high in the day 7 of neural differentiation. This is in line with other studies which specified Sox1/SOX1 as an early responder to neural inducing signals and one of the early markers of neural induction [50–52]. Regarding the methylation status of *SOX1* gene, data are limited to various types of cancers [53–55], while for neural differentiation experimental data are lacking. Thus, we proceeded with the MSP analysis of the methylation status of *SOX1* gene promoter during the neural differentiation of NT2/D1 cells. We detected products obtained with primer set corresponding to unmethylated DNA, while the products obtained with primer set corresponding to methylated DNA were absent, as shown in Fig 6A. These results indicated that *SOX1* promoter has a low methylation level in both undifferentiated NT2/D1 cells, as well as during the following days of RA-induced neural differentiation, similar to methylation profiles obtained for *SOX3* promoter.

**Fig 7:**
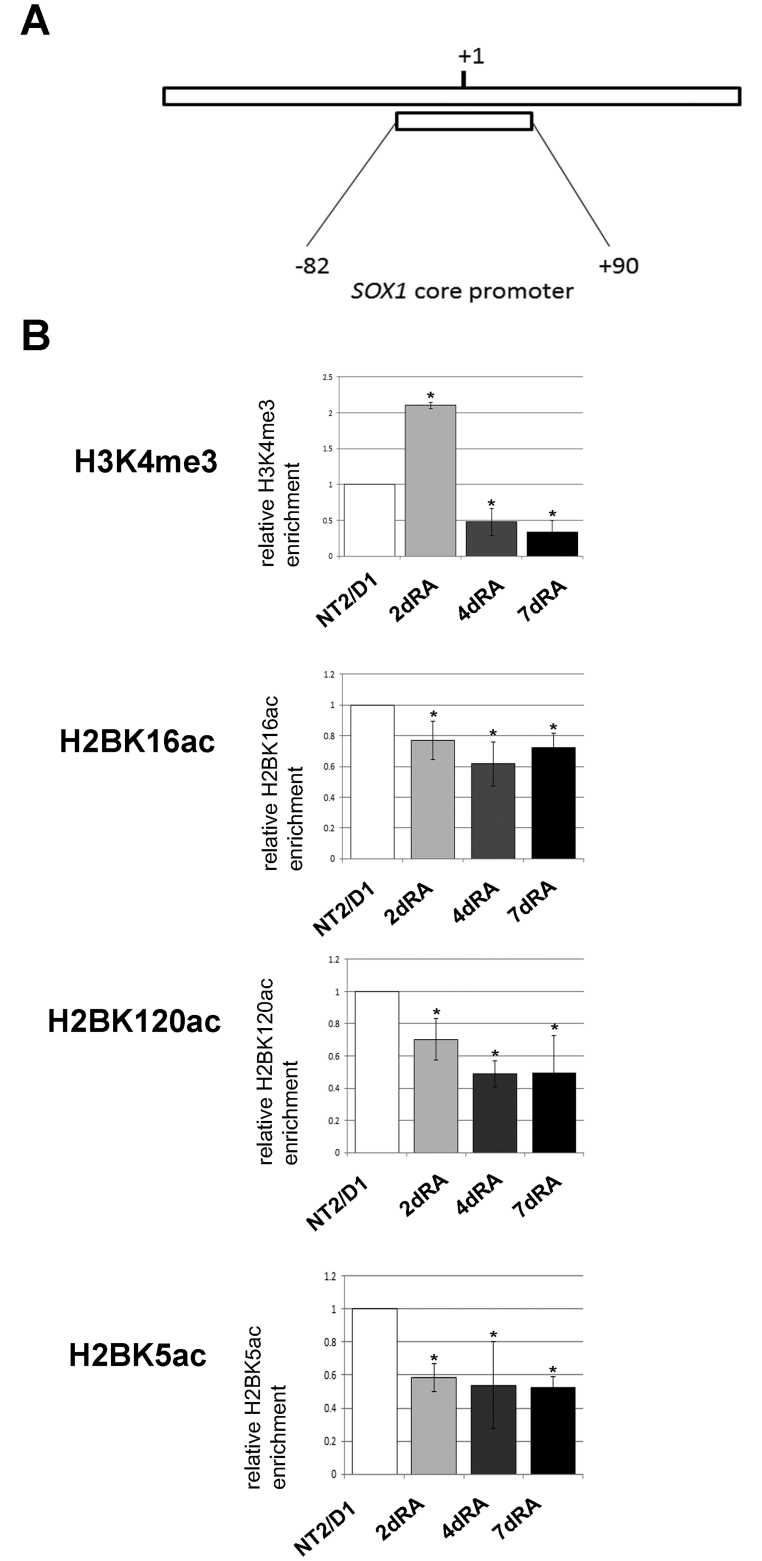
ChIP-qPCR analysis of the *SOX1* core promoter. (A) Schematic representation of the human *SOX1* core promoter analyzed by ChIP. The positions of the region relative to TSS are indicated. (B) ChIP-qPCR results for the indicated histone modifications. The enrichment was calculated relative to Flag and normalized against H3 or H2B. In comparative experiments, the enrichment in undifferentiated cells was assigned the value 1 and other samples were normalized to this value. Each ChIP experiment was repeated three times (biological replicates) followed by duplicate qPCR reactions. Results are presented as the mean ± S.D., *P<0.05.

The analyses of histone PTMs revealed increase in H3K4me3 abundance on the promoter of *SOX1* at day 2 of RA induction (Fig 7B). In following days of RA treatment we observed slight drop in the enrichment of H3K4me3, indicating that this histone mark is not in correlation with the detected transcriptional activation of *SOX1*. As for H2B modifications, prominent decline in H2BK16ac, H2BK120ac and H2BK5ac levels accompanied RA-induced neural differentiation of the cells (Fig 7B). These data suggest that selected H2B acetyl marks do not contribute to the enhanced SOX1 expression following RA induction.

### Analysis of human *SOX2* gene expression and histone modifications profiles during the early phases of neural differentiation of NT2/D1 cells

**Fig 8:**
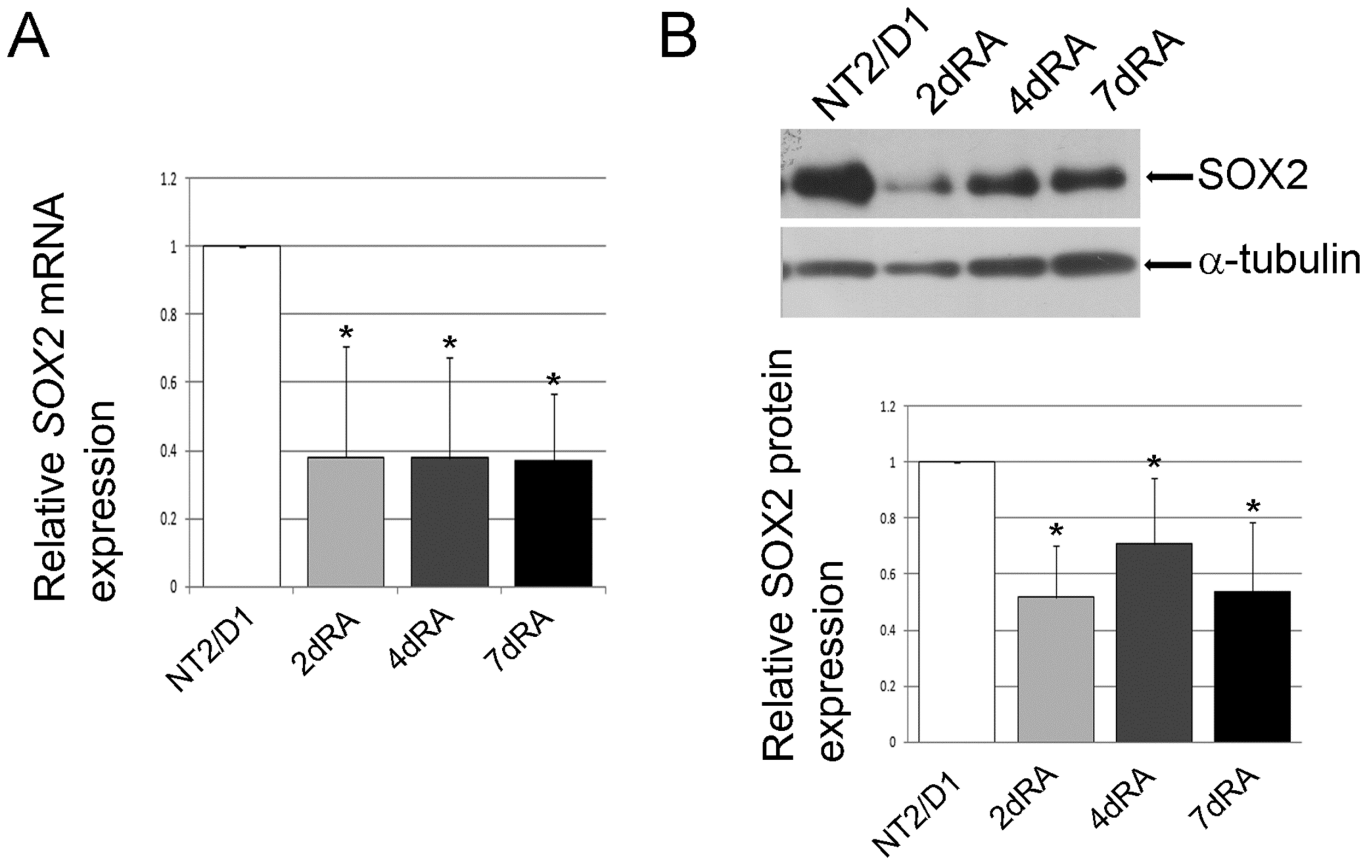
*SOX2* is down-regulated during early phases of RA induced neural differentiation of NT2/D1 cells. (A) Real-time PCR analysis of *SOX2* gene expression in untreated and RA treated NT2/D1 cells (2, 4 and 7 days of induction). Data were normalized by the amount of *GAPDH* mRNA and presented relative to the corresponding value for untreated cells, and are means ± S.D., *P<0.05 from triplicate data. (B) Western blot analysis of SOX2 protein in whole cell lysates of untreated and NT2/D1 cells treated with RA for 2, 4 and 7 days. SOX2 protein quantities were expressed relative to untreated NT2/D1 cells (set at 1) and presented as the mean ± S.D. of at least three independent experiments, *P<0.05. α-tubulin was used as loading control. Representative blots are shown.

We analyzed the expression pattern of *SOX2*/SOX2 mRNA and protein levels in differentiating NT2/D1 cells. As shown in Fig 8, we detected a significant drop in *SOX2*/SOX2 mRNA and protein levels 2 days after exposure to RA, with no further decline in the following days (Fig 8A and 8B). This is consistent with the exit from pluripotency and activation of the neural program [35]. It also coincides with the increase in *SOX3* expression, indicating that *SOX2* and *SOX3* are differentially regulated. Numerous studies have demonstrated that the regulatory regions of *SOX2* are nonmethylated during the course of neural differentiation of embryonal carcinoma cells [23,56] suggesting that the changes of *SOX2* expression during neural differentiation are independent of DNA methylation.

**Fig 9:**
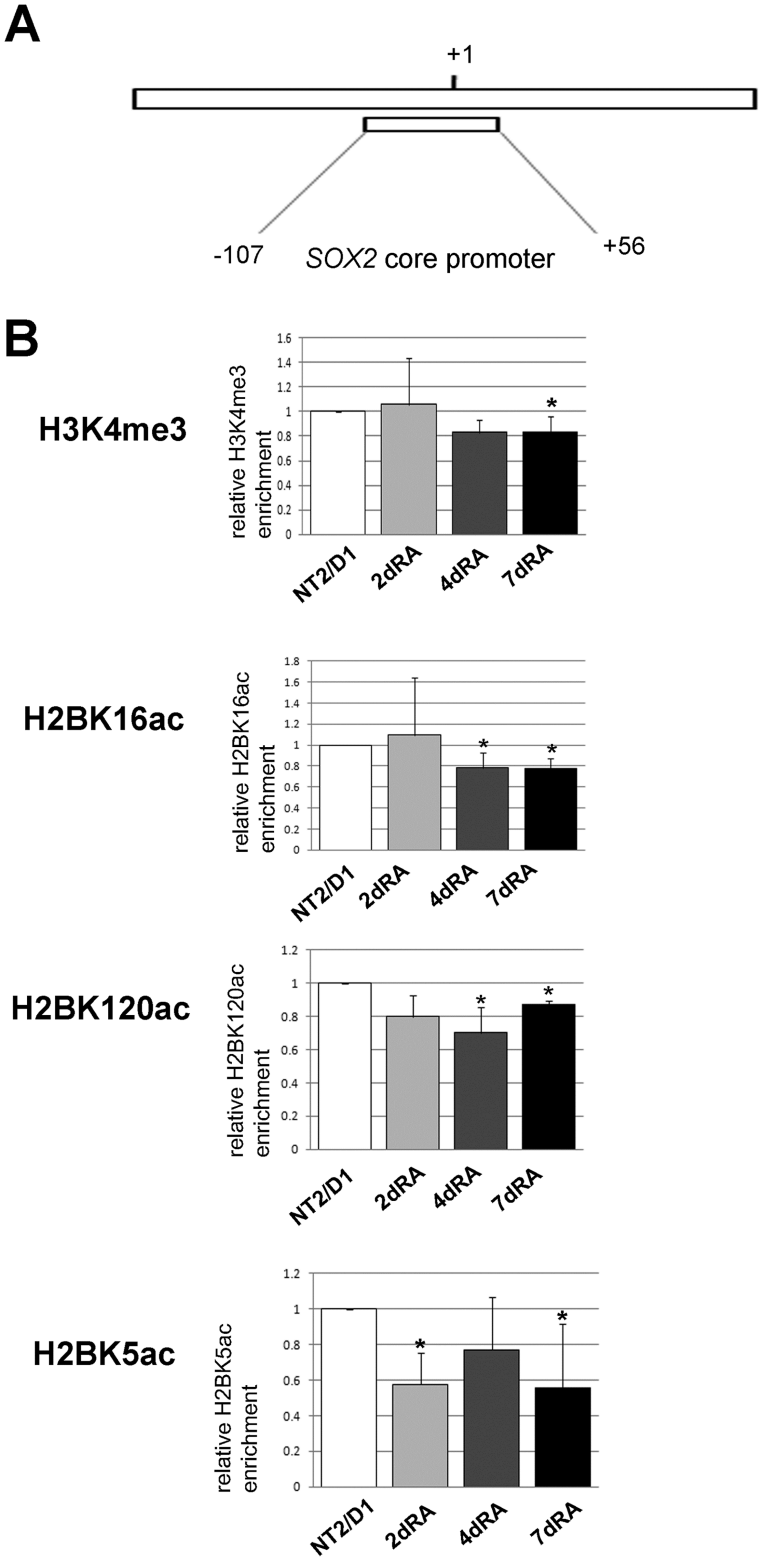
ChIP-qPCR analysis of the *SOX2* core promoter. Schematic representation of the human *SOX2* core promoter analyzed by ChIP. The positions of the region relative to TSS are indicated. (B) ChIP-qPCR results for the indicated histone modifications. The enrichment was calculated relative to Flag and normalized against H3 or H2B. In comparative experiments, the enrichment in undifferentiated cells was assigned the value 1 and other samples were normalized to this value. Each ChIP experiment was repeated three times (biological replicates) followed by duplicate qPCR reactions. Results are presented as the mean ± S.D., *P<0.05.

As for histone PTMs, we detected high level of H3K4me3 on the human *SOX2* promoter, consistent with the previous studies performed in mouse and human ESCs [57–59], as well as high levels of H2BK16ac, H2BK120ac and H2BK5ac. Following treatment of NT2/D1 cells with RA, we observed a slight decrease in H3K4me3, H2BK16ac and H2BK120ac (Fig 9). The most prominent change was in H2BK5ac, which declined on day 2 of differentiation (Fig 9). This coincides with the decrease of *SOX2* expression (Fig 8), suggesting that deacetylation of H2BK5 is one of the marks associated to the response of the *SOX2* promoter to the RA. The *SOX2* promoter is showing an opposite profile of H2BK5ac compared to *SOX3* at day 2, which implies that these two genes have different epigenetic regulation (Figs 5 and 9).

### Bioinformatic analyses of human *SOXB1* genes expression and histone modifications profiles in H1-ESCs and H1-derived neural progenitors

In order to assess expression and histone PTMs profiles of *SOXB1* genes in another model system, we performed bioinformatics analysis of genome-wide data available from http://www.roadmapepigenomics.org/ for H1-ESCs and H1-derived neural progenitors. Data are presented in S1 Fig. Furthermore, expression data obtained in the present study are in line with RNA-Seq analyses of expression of *SoxB1* mRNAs during RA-induced neuronal differentiation of mESCs [60].

## Discussion

Despite the growing amount of data regarding the regulation of the human *SOX3* gene expression and activity in pluripotent cells and their differentiated progeny, reports concerning the epigenetic mechanisms of its regulation during the process of differentiation are limited. Lindeman et al. revealed that regulatory sequences of the *Sox3* zebrafish orthologue are methylated and low in positive histone PTMs in somatic cells, compared to midblastula transition (MBT) cells, characterized by high expression of *Sox3*, lack of DNA methylation and positive histone PTMs [61]. Epigenetic regulation of *Sox3* has also been discussed as part of study by Azuara et al. [59] showing that markers of active and repressive chromatin are simultaneously present at silent tissue-specific genes, such as *Sox3*, in mESCs. Finally, hypermethylation of *SOX3* was detected in chronic lymphocyte leukemia [62].

Embryonal carcinoma cell line NT2/D1 provided us with a valuable model system to study the epigenetic state of *SOX3* gene in pluripotent cells as well as in cells responding to neural differentiation-inducing stimuli. Molecular events during RA induced differentiation of NT2/D1 cells reflect the steps in the development of human brain [63]. We demonstrated that the initial phases of RA treatment are critical for the transcriptional activity of *SOX3* [17,32,35]. Two days after RA introduction, we detected a significant increase in SOX3 mRNA and protein, thereafter, downregulation ensued. This profile resembles the one previously detected in the chicken and mouse neuroepithelium during early phases of neural induction, with transient upregulation of *Sox3* gene [64–66].

Despite low SOX3 expression levels in undifferentiated cells, the *SOX3* promoter is hypomethylated. This hypomethylation persists through neural differentiation induced by RA. Similar results were obtained for the *SOX1* promoter, which is also hypomethylated during the first 7 days of neural differentiation of NT2/D1 cells. While methylation of CpG–rich promoters is considered a hallmark of silent genes, nonmethylated promoters are not necessarily associated with active transcription [67,68]. Rather, hypomethylation allows a permissive platform on which other mechanisms mediating the recruitment of different transcription factors act [67]. Some of the germline- and pluripotency-associated genes with hypomethylated promoters, and low expression levels in stem cells, acquire methylation during lineage commitment [69]. Moreover, the regulatory regions of *SOX2* which are unmethylated in undifferentiated NT2/D1 cells become methylated in neurons, with no detectable SOX2 expression [23]. At the same time, differentiation-induced repression of SOX2 is paralleled by shift from H3K4me3 to colocalization of both H3K4me3 and H3K27me3, as demonstrated in ES cells [58].

We demonstrate that H3K4me3 on the core promoter and upstream of TSS is associated with transcriptional induction of *SOX3* gene. This finding is supported by the study by Lauberth et al. showing that H3K4me3 facilitates TFIID recruitment and enhances preinitiation compex (PIC) assembly, thus promoting transcription [70]. Many promoters in ES cells are positive for both H3K27me3 and H3K4me3 and there is evidence indicating that *SOX3* and *SOX1* are among the genes with bivalent promoters. In the study by Mikkelsen et al., *Sox3* was one of the genes with bivalent promoter in mES cells, resolved into H3K4me3^+^ in lineage committed NP cells, while in mouse embryonic fibroblasts (MEFs) it is devoid of any histone marks [57]. In the same study, *Sox1* was found to be bivalent in both mES and NP cells, and H3K4me3^-^/H3K27me3^+^ in MEFs [57]. Furthermore, in the study by Pan et al. *SOX1* was found to be marked by both H3K4 and H3K27 trimethylation during RA- and BMP4- induced differentiation of ES cells [58]. This state of *Sox3* and *Sox1* is typical for high CpG content promoters of genes with key roles in the development [57]. Further ChIP analyses are necessary in order to test this hypothesis.

Interestingly, activation of *SOX3* at day 2 was not accompanied by an increase in H2B acetylation. There is limited literature data regarding the contribution of H2B acetylation in regulation of transcriptional regulation: a study by Kurdistani et al. demonstrated negative correlation of H2B acetyl marks with transcription in *Saccharomyces cerevisiae* [71]. Analyses of histone modification patterns in yeast suggested that acetylation of H2A and H2B has cumulative transcriptional effects together with H3 and H4 acetylations [72,73]. The lack of increase in H2B acetylation at the time point with the highest SOX3 expression implies that upregulation of the *SOX3* gene upon RA treatment is associated to other histone marks. Nevertheless, substantial depletion of all three H2B acetyl marks on the promoter of *SOX3* at day 7 coincides with the reduced expression of *SOX3* gene. These findings suggest that H2BK5ac, H2BK16ac and H2BK120ac at the core promoter of *SOX3* gene contribute to the regulation of *SOX3* gene expression, primarily at the end of the first week of neural differentiation of NT2/D1 cells, when they interact coordinately with other deposited histone marks and transcription apparatus or transcription factors. It would be interesting to evaluate chromatin marks on other potential regulatory regions of *SOX3* gene. For example, recent analysis has revealed that distal enhancers of numerous genes are targeted by NF-Y, a transcriptional regulator of *SOX3* gene and transcription factor with nucleosome-like properties that mediates establishment of permissive chromatin modifications [14,74,75]. Further analysis could reveal the existence and function of corresponding elements at distal regions of human *SOX3* gene.

One of the interesting findings emerging from this study is that *SOXB1* genes are differentially regulated in the initial phases of neural differentiation. Based on the presented data, early phase of neural differentiation of NT2/D1 cells could be divided into two stages. First stage falls within the first 2 days of RA induction and is characterized by the increase in SOX3 and reduction in SOX2 expression levels, whereas SOX1 remains unchanged. Second stage (days 4 and 7) is accompanied by the reduction in both SOX2 and SOX3 levels, while SOX1 is upregulated. In the first stage, H3K4me3 profile of the *SOX2* promoter is not strongly correlated with the decline in the expression of *SOX2*, as opposed to *SOX3* whose upregulation at day 2 is likely operated by H3K4me3. This is in concordance with the findings by Barrand et al. indicating that H3K4me3 is associated with *SOX2* promoter in different cell types, regardless of *SOX2* expression [56]. Moreover, H2BK5ac abundance at day 2 of RA induction is considerably lower for *SOX2* than for *SOX3* promoter, providing an additional proof of different states of *SOX2* and *SOX3* promoters in cells undergoing differentiation. As for *SOX1* promoter, we detected raise in H3K4me3 at day 2, preceeding the actual increase in SOX1 expression levels at day 4 and 7 of RA induction.

In the second stage, at day 7 we detected significant decrease in all analyzed histone PTMs at promoters of *SOX1*, *SOX2* and *SOX3* genes. These implies that same epigenetic mechanisms could contribute to the observed reduction in SOX2 and SOX3 expression levels at later stages of neural differentiation. Conversely, detected decrease in H3K4me3 and H2B acetyl marks at *SOX1* promoter suggests that these activating histone modifications do not have considerable role in transcriptional activation of *SOX1*. Therefore, other mechanisms such as a depletion of repressive mark H3K27me3 or changes in TFs activity and signaling pathways could be attributable to changes in *SOX1* expression during the neural differentiation. It would be significant to analyze epigenetic mechanisms acting on *SOXB1* genes in later stages of neural differentiation, as well as in cells undergoing the process of dedifferentiation, as this could have impact on our understanding of the exact roles of *SOXB1* genes in pluripotentcy maintenance and on improvement of guided differentiation protocols.

Regarding the other mechanisms implicated in the regulation of *SOXB1* genes, we have previously described the modulation of *SOX2* and *SOX3* gene expression during the early phases of neural induction of NT2/D1 cells by RA [35]. Also, we have performed extensive functional characterization of the regulatory regions within human *SOX3* promoter involved in RA-responsiveness [12,14,15,17]. We have described several elements, including an atypical RAR/RXRα response element (RE), located at position -259 to -154 [15], and a DR-3-like RXR RE, positioned -68 to -54 relative to TSS [17]. We demonstrated the involvement of several transcription factors (NF-Y, PBX1 and MEIS1) in the regulation of expression of this gene during initial 48h of neural induction [14,15]. In addition, we analyzed whether some aspects of transcriptional regulation are preserved between human *SOX2* and *SOX3* genes during first 48h of RA induction [76]. Among many similarities (activation by Sp1, MAZ, PBX1, MEIS1 and liganded RXR) we observed that TGIF acted as a transcriptional repressor of the *SOX3* gene, while no significant effect of this TF on the *SOX2* expression has been observed. Also, in contrast to significant impact of NF-Y on RA-induced activation of *SOX3*, this TF had only mild effect on *SOX2* expression in RA-induced NT2/D1 cells [76]. This observation is in concordance with the observation of Wiebe et al. that *SOX2* promoter activity is down-regulated upon RA induction of mouse F9 EC cells [77]. Indeed, our results confirmed speculations of Wiebe et al. that epigenetic mechanisms could contribute to the silencing of *Sox2* promoter upon differentiation of EC cells [77]. Apart from these nearby regions expression of both genes are regulated by far distance enhancers [27,78–80] and multiple regulatory mechanisms (e.g signaling pathways) [81–87]. It is important to emphasize that SOX2 expression is also modulated through activity of miRNA and ncRNA. miRNA-134 and miRNA-145 repress SOX2 expression by targeting its coding region in mES cells and the 3′-UTR in hES cells, respectively [88,89]. Also, it has been postulated that ncRNA SOX2OT (SOX2 overlapping transcript) participate in *SOX2* transcriptional regulation acting as an enhancer [90]. As for the *SOX1*, there are several studies demonstrating the roles of signaling pathways in *SOX1* gene regulation [83,86,91,92], while data regarding TFs involvement in the regulation of this gene expression during the neural differentiation are lacking.

In summary, we provide a first map of the epigenetic landscape of *SOX3* in pluripotent cells and during the early phases of neural differentiation. We found *SOX3* gene to be non methylated from undifferentiated NT2/D1 to cells committed towards neural lineage. Furthermore, we presented data regarding epigenetic mechanisms acting on *SOX2* and *SOX1* genes during the initial phases of neural differentiation, pointing out to potential similarities and differences in the epigenetic control of *SOXB1* genes. These findings could contribute to the elucidation of complex events during neural differentiation and ultimately provide better means for the development of therapies based on the use of epigenetic-modifying drugs.

## Supporting information

S1 Fig Bioinformatic analyses of expression and histone PTMs of *SOXB1* genes in H1-ESCs and H1-derived neural progenitors. Raw data were retrieved from http://www.roadmapepigenomics.org/ and converted in bigwig files through Galaxy tool followed by the visualization in UCSC genome browser.
(PPTX)

## References

[1] Bergsland M, Ramskold D, Zaouter C, Klum S, Sandberg R, Muhr J (2011) Sequentially acting Sox transcription factors in neural lineage development. Genes Dev 25: 2453–2464. doi: 10.1101/gad.176008.111 22085726 10.1101/gad.176008.111 PMC3243056

[2] Stevanovic M, Lovell-Badge R, Collignon J, Goodfellow PN (1993) SOX3 is an X-linked gene related to SRY. Hum Mol Genet 2: 2013–2018. 8111369 10.1093/hmg/2.12.2013

[3] Wegner M (2010) All purpose Sox: The many roles of Sox proteins in gene expression. Int J Biochem Cell Biol 42: 381–390. doi: 10.1016/j.biocel.2009.07.006 19631281 10.1016/j.biocel.2009.07.006

[4] Bylund M, Andersson E, Novitch BG, Muhr J (2003) Vertebrate neurogenesis is counteracted by Sox1-3 activity. Nat Neurosci 6: 1162–1168. doi: 10.1038/nn1131 14517545 10.1038/nn1131

[5] Graham V, Khudyakov J, Ellis P, Pevny L (2003) SOX2 functions to maintain neural progenitor identity. Neuron 39: 749–765. 12948443 10.1016/s0896-6273(03)00497-5

[6] Avilion AA, Nicolis SK, Pevny LH, Perez L, Vivian N, Lovell-Badge R (2003) Multipotent cell lineages in early mouse development depend on SOX2 function. Genes Dev 17: 126–140. doi: 10.1101/gad.224503 12514105 10.1101/gad.224503 PMC195970

[7] Rogers N, Cheah PS, Szarek E, Banerjee K, Schwartz J, Thomas P (2013) Expression of the murine transcription factor SOX3 during embryonic and adult neurogenesis. Gene Expr Patterns 13: 240–248. doi: 10.1016/j.gep.2013.04.004 23665444 10.1016/j.gep.2013.04.004

[8] McAninch D, Thomas P (2014) Identification of Highly Conserved Putative Developmental Enhancers Bound by SOX3 in Neural Progenitors Using ChIP-Seq. PLoS One 9: e113361 doi: 10.1371/journal.pone.0113361 25409526 10.1371/journal.pone.0113361 PMC4237438

[9] Rogers N, McAninch D, Thomas P (2014) Dbx1 is a direct target of SOX3 in the spinal cord. PLoS One 9: e95356 doi: 10.1371/journal.pone.0095356 24751947 10.1371/journal.pone.0095356 PMC3994032

[10] Wang Z, Oron E, Nelson B, Razis S, Ivanova N (2012) Distinct lineage specification roles for NANOG, OCT4, and SOX2 in human embryonic stem cells. Cell Stem Cell 10: 440–454. doi: 10.1016/j.stem.2012.02.016 22482508 10.1016/j.stem.2012.02.016

[11] Nakagawa M, Koyanagi M, Tanabe K, Takahashi K, Ichisaka T, Aoi T, et al (2008) Generation of induced pluripotent stem cells without Myc from mouse and human fibroblasts. Nat Biotechnol 26: 101–106. doi: 10.1038/nbt1374 18059259 10.1038/nbt1374

[12] Kovacevic Grujicic N, Mojsin M, Krstic A, Stevanovic M (2005) Functional characterization of the human SOX3 promoter: identification of transcription factors implicated in basal promoter activity. Gene 344: 287–297. doi: 10.1016/j.gene.2004.11.006 15656994 10.1016/j.gene.2004.11.006

[13] Kovacevic-Grujicic N, Mojsin M, Popovic J, Petrovic I, Topalovic V, Stevanovic M (2014) Cyclic AMP response element binding (CREB) protein acts as a positive regulator of SOX3 gene expression in NT2/D1 cells. BMB Rep 47: 197–202. doi: 10.5483/BMBRep.2014.47.4.084 24257117 10.5483/BMBRep.2014.47.4.084 PMC4163894

[14] Krstic A, Mojsin M, Stevanovic M (2007) Regulation of SOX3 gene expression is driven by multiple NF-Y binding elements. Arch Biochem Biophys 467: 163–173. doi: 10.1016/j.abb.2007.08.029 17910945 10.1016/j.abb.2007.08.029

[15] Mojsin M, Grujicic NK, Nikcevic G, Krstic A, Savic T, Stevanovic M (2006) Mapping of the RXRalpha binding elements involved in retinoic acid induced transcriptional activation of the human SOX3 gene. Neurosci Res 56: 409–418. doi: 10.1016/j.neures.2006.08.010 17005281 10.1016/j.neures.2006.08.010

[16] Mojsin M, Stevanovic M (2010) PBX1 and MEIS1 up-regulate SOX3 gene expression by direct interaction with a consensus binding site within the basal promoter region. Biochem J 425: 107–116. 10.1042/BJ20090694 19799567

[17] Nikcevic G, Savic T, Kovacevic-Grujicic N, Stevanovic M (2008) Up-regulation of the SOX3 gene expression by retinoic acid: characterization of the novel promoter-response element and the retinoid receptors involved. J Neurochem 107: 1206–1215. doi: 10.1111/j.1471-4159.2008.05670.x 18786169 10.1111/j.1471-4159.2008.05670.x

[18] Mohn F, Schubeler D (2009) Genetics and epigenetics: stability and plasticity during cellular differentiation. Trends Genet 25: 129–136. doi: 10.1016/j.tig.2008.12.005 19185382 10.1016/j.tig.2008.12.005

[19] Ang YS, Gaspar-Maia A, Lemischka IR, Bernstein E (2011) Stem cells and reprogramming: breaking the epigenetic barrier? Trends Pharmacol Sci 32: 394–401. doi: 10.1016/j.tips.2011.03.002 21621281 10.1016/j.tips.2011.03.002 PMC3128683

[20] Olynik BM, Rastegar M (2012) The genetic and epigenetic journey of embryonic stem cells into mature neural cells. Front Genet 3: 81 doi: 10.3389/fgene.2012.00081 22629283 10.3389/fgene.2012.00081 PMC3355330

[21] Deb-Rinker P, Ly D, Jezierski A, Sikorska M, Walker PR (2005) Sequential DNA methylation of the Nanog and Oct-4 upstream regions in human NT2 cells during neuronal differentiation. J Biol Chem 280: 6257–6260. doi: 10.1074/jbc.C400479200 15615706 10.1074/jbc.C400479200

[22] Nettersheim D, Biermann K, Gillis AJ, Steger K, Looijenga LH, Schorle H (2011) NANOG promoter methylation and expression correlation during normal and malignant human germ cell development. Epigenetics 6: 114–122. doi: 10.4161/epi.6.1.13433 20930529 10.4161/epi.6.1.13433 PMC3052918

[23] Sikorska M, Sandhu JK, Deb-Rinker P, Jezierski A, Leblanc J, Charlebois C, et al (2008) Epigenetic modifications of SOX2 enhancers, SRR1 and SRR2, correlate with in vitro neural differentiation. J Neurosci Res 86: 1680–1693. doi: 10.1002/jnr.21635 18293417 10.1002/jnr.21635

[24] Andrews PW (1984) Retinoic acid induces neuronal differentiation of a cloned human embryonal carcinoma cell line in vitro. Dev Biol 103: 285–293. 6144603 10.1016/0012-1606(84)90316-6

[25] Li LC, Dahiya R (2002) MethPrimer: designing primers for methylation PCRs. Bioinformatics 18: 1427–1431. 12424112 10.1093/bioinformatics/18.11.1427

[26] Rank G, Cerruti L, Simpson RJ, Moritz RL, Jane SM, Zhao Q (2010) Identification of a PRMT5-dependent repressor complex linked to silencing of human fetal globin gene expression. Blood 116: 1585–1592. doi: 10.1182/blood-2009-10-251116 20495075 10.1182/blood-2009-10-251116 PMC2938845

[27] Brunelli S, Silva Casey E, Bell D, Harland R, Lovell-Badge R (2003) Expression of Sox3 throughout the developing central nervous system is dependent on the combined action of discrete, evolutionarily conserved regulatory elements. Genesis 36: 12–24. doi: 10.1002/gene.10193 12748963 10.1002/gene.10193

[28] Andrews PW, Nudelman E, Hakomori S, Fenderson BA (1990) Different patterns of glycolipid antigens are expressed following differentiation of TERA-2 human embryonal carcinoma cells induced by retinoic acid, hexamethylene bisacetamide (HMBA) or bromodeoxyuridine (BUdR). Differentiation 43: 131–138. 2373286 10.1111/j.1432-0436.1990.tb00439.x

[29] Goodfellow CE, Graham SE, Dragunow M, Glass M (2011) Characterization of NTera2/D1 cells as a model system for the investigation of cannabinoid function in human neurons and astrocytes. J Neurosci Res 89: 1685–1697. doi: 10.1002/jnr.22692 21674570 10.1002/jnr.22692

[30] Pleasure SJ, Lee VM (1993) NTera 2 cells: a human cell line which displays characteristics expected of a human committed neuronal progenitor cell. J Neurosci Res 35: 585–602. doi: 10.1002/jnr.490350603 8411264 10.1002/jnr.490350603

[31] Hara K, Yasuhara T, Maki M, Matsukawa N, Masuda T, Yu SJ, et al (2008) Neural progenitor NT2N cell lines from teratocarcinoma for transplantation therapy in stroke. Prog Neurobiol 85: 318–334. doi: 10.1016/j.pneurobio.2008.04.005 18514379 10.1016/j.pneurobio.2008.04.005

[32] Nikcevic G, Kovacevic-Grujicic N, Mojsin M, Krstic A, Savic T, Stevanovic M (2011) Regulation of the SOX3 gene expression by retinoid receptors. Physiol Res 60 Suppl 1: S83–91. 21777018 10.33549/physiolres.932184

[33] Popovic J, Stanisavljevic D, Schwirtlich M, Klajn A, Marjanovic J, Stevanovic M (2014) Expression analysis of SOX14 during retinoic acid induced neural differentiation of embryonal carcinoma cells and assessment of the effect of its ectopic expression on SOXB members in HeLa cells. PLoS One 9: e91852 doi: 10.1371/journal.pone.0091852 24637840 10.1371/journal.pone.0091852 PMC3956720

[34] Topalovic V, Schwirtlich M, Stevanovic M, Mojsin M (2017) Histone Modifications on the Promoters of Human OCT4 and NANOG Genes at the Onset of Neural Differentiation of NT2/D1 Cells. Biochemistry (Mosc) 82: 715–722. 28601081 10.1134/S0006297917060086

[35] Stevanovic M (2003) Modulation of SOX2 and SOX3 gene expression during differentiation of human neuronal precursor cell line NTERA2. Mol Biol Rep 30: 127–132. 12841584 10.1023/a:1023961009869

[36] Shahryari A, Rafiee MR, Fouani Y, Oliae NA, Samaei NM, Shafiee M, et al (2014) Two novel splice variants of SOX2OT, SOX2OT-S1, and SOX2OT-S2 are coupregulated with SOX2 and OCT4 in esophageal squamous cell carcinoma. Stem Cells 32: 126–134. doi: 10.1002/stem.1542 24105929 10.1002/stem.1542

[37] Liao R, Mizzen CA (2017) Site-specific regulation of histone H1 phosphorylation in pluripotent cell differentiation. Epigenetics Chromatin 10: 29 doi: 10.1186/s13072-017-0135-3 28539972 10.1186/s13072-017-0135-3 PMC5440973

[38] Saunders A, Faiola F, Wang J (2013) Concise review: pursuing self-renewal and pluripotency with the stem cell factor Nanog. Stem Cells 31: 1227–1236. doi: 10.1002/stem.1384 23653415 10.1002/stem.1384 PMC3706551

[39] Pierani A, Moran-Rivard L, Sunshine MJ, Littman DR, Goulding M, Jessell TM (2001) Control of interneuron fate in the developing spinal cord by the progenitor homeodomain protein Dbx1. Neuron 29: 367–384. 11239429 10.1016/s0896-6273(01)00212-4

[40] Tost J, Dunker J, Gut IG (2003) Analysis and quantification of multiple methylation variable positions in CpG islands by Pyrosequencing. Biotechniques 35: 152–156. 12866415 10.2144/03351md02

[41] Santi DV, Norment A, Garrett CE (1984) Covalent bond formation between a DNA-cytosine methyltransferase and DNA containing 5-azacytosine. Proc Natl Acad Sci U S A 81: 6993–6997. 6209710 10.1073/pnas.81.22.6993 PMC392062

[42] Taylor SM, Jones PA (1982) Mechanism of action of eukaryotic DNA methyltransferase. Use of 5-azacytosine-containing DNA. J Mol Biol 162: 679–692. 6187927 10.1016/0022-2836(82)90395-3

[43] Musch T, Oz Y, Lyko F, Breiling A (2010) Nucleoside drugs induce cellular differentiation by caspase-dependent degradation of stem cell factors. PLoS One 5: e10726 doi: 10.1371/journal.pone.0010726 20502711 10.1371/journal.pone.0010726 PMC2873290

[44] Cruickshank MN, Besant P, Ulgiati D (2010) The impact of histone post-translational modifications on developmental gene regulation. Amino Acids 39: 1087–1105. doi: 10.1007/s00726-010-0530-6 20204433 10.1007/s00726-010-0530-6

[45] Grunstein M (1997) Histone acetylation in chromatin structure and transcription. Nature 389: 349–352. doi: 10.1038/38664 9311776 10.1038/38664

[46] Legube G, Trouche D (2003) Regulating histone acetyltransferases and deacetylases. EMBO Rep 4: 944–947. doi: 10.1038/sj.embor.embor941 14528264 10.1038/sj.embor.embor941 PMC1326399

[47] Okitsu CY, Hsieh JC, Hsieh CL (2010) Transcriptional activity affects the H3K4me3 level and distribution in the coding region. Mol Cell Biol 30: 2933–2946. doi: 10.1128/MCB.01478-09 20404096 10.1128/MCB.01478-09 PMC2876678

[48] Jonkers I, Kwak H, Lis JT (2014) Genome-wide dynamics of Pol II elongation and its interplay with promoter proximal pausing, chromatin, and exons. Elife 3: e02407 doi: 10.7554/eLife.02407 24843027 10.7554/eLife.02407 PMC4001325

[49] Zentner GE, Henikoff S (2013) Regulation of nucleosome dynamics by histone modifications. Nat Struct Mol Biol 20: 259–266. doi: 10.1038/nsmb.2470 23463310 10.1038/nsmb.2470

[50] Pevny LH, Sockanathan S, Placzek M, Lovell-Badge R (1998) A role for SOX1 in neural determination. Development 125: 1967–1978. 9550729 10.1242/dev.125.10.1967

[51] Abranches E, Silva M, Pradier L, Schulz H, Hummel O, Henrique D, et al (2009) Neural differentiation of embryonic stem cells in vitro: a road map to neurogenesis in the embryo. PLoS One 4: e6286 doi: 10.1371/journal.pone.0006286 19621087 10.1371/journal.pone.0006286 PMC2709448

[52] Baharvand H, Mehrjardi NZ, Hatami M, Kiani S, Rao M, Haghighi MM (2007) Neural differentiation from human embryonic stem cells in a defined adherent culture condition. Int J Dev Biol 51: 371–378. doi: 10.1387/ijdb.72280hb 17616926 10.1387/ijdb.72280hb

[53] Guan Z, Zhang J, Wang J, Wang H, Zheng F, Peng J, et al (2014) SOX1 down-regulates beta-catenin and reverses malignant phenotype in nasopharyngeal carcinoma. Mol Cancer 13: 257 doi: 10.1186/1476-4598-13-257 25427424 10.1186/1476-4598-13-257 PMC4326525

[54] Lin YW, Tsao CM, Yu PN, Shih YL, Lin CH, Yan MD (2013) SOX1 suppresses cell growth and invasion in cervical cancer. Gynecol Oncol 131: 174–181. doi: 10.1016/j.ygyno.2013.07.111 23927962 10.1016/j.ygyno.2013.07.111

[55] Tsao CM, Yan MD, Shih YL, Yu PN, Kuo CC, Lin WC, et al (2012) SOX1 functions as a tumor suppressor by antagonizing the WNT/beta-catenin signaling pathway in hepatocellular carcinoma. Hepatology 56: 2277–2287. doi: 10.1002/hep.25933 22767186 10.1002/hep.25933

[56] Barrand S, Collas P (2010) Chromatin states of core pluripotency-associated genes in pluripotent, multipotent and differentiated cells. Biochem Biophys Res Commun 391: 762–767. doi: 10.1016/j.bbrc.2009.11.134 19944068 10.1016/j.bbrc.2009.11.134

[57] Mikkelsen TS, Ku M, Jaffe DB, Issac B, Lieberman E, Giannoukos G, et al (2007) Genome-wide maps of chromatin state in pluripotent and lineage-committed cells. Nature 448: 553–560. doi: 10.1038/nature06008 17603471 10.1038/nature06008 PMC2921165

[58] Pan G, Tian S, Nie J, Yang C, Ruotti V, Wei H, et al (2007) Whole-genome analysis of histone H3 lysine 4 and lysine 27 methylation in human embryonic stem cells. Cell Stem Cell 1: 299–312. doi: 10.1016/j.stem.2007.08.003 18371364 10.1016/j.stem.2007.08.003

[59] Azuara V, Perry P, Sauer S, Spivakov M, Jorgensen HF, John RM, et al (2006) Chromatin signatures of pluripotent cell lines. Nat Cell Biol 8: 532–538. doi: 10.1038/ncb1403 16570078 10.1038/ncb1403

[60] Terranova C, Narla ST, Lee YW, Bard J, Parikh A, Stachowiak EK, et al (2015) Global Developmental Gene Programing Involves a Nuclear Form of Fibroblast Growth Factor Receptor-1 (FGFR1). PLoS One 10: e0123380 doi: 10.1371/journal.pone.0123380 25923916 10.1371/journal.pone.0123380 PMC4414453

[61] Lindeman LC, Winata CL, Aanes H, Mathavan S, Alestrom P, Collas P (2010) Chromatin states of developmentally-regulated genes revealed by DNA and histone methylation patterns in zebrafish embryos. Int J Dev Biol 54: 803–813. doi: 10.1387/ijdb.103081ll 20336603 10.1387/ijdb.103081ll

[62] Rahmatpanah FB, Carstens S, Hooshmand SI, Welsh EC, Sjahputera O, Taylor KH, et al (2009) Large-scale analysis of DNA methylation in chronic lymphocytic leukemia. Epigenomics 1: 39–61. doi: 10.2217/epi.09.10 20495622 10.2217/epi.09.10 PMC2872502

[63] Przyborski SA, Morton IE, Wood A, Andrews PW (2000) Developmental regulation of neurogenesis in the pluripotent human embryonal carcinoma cell line NTERA-2. Eur J Neurosci 12: 3521–3528. 11029621 10.1046/j.1460-9568.2000.00230.x

[64] Collignon J, Sockanathan S, Hacker A, Cohen-Tannoudji M, Norris D, Rastan S, et al (1996) A comparison of the properties of Sox-3 with Sry and two related genes, Sox-1 and Sox-2. Development 122: 509–520. 8625802 10.1242/dev.122.2.509

[65] Pevny LH, Lovell-Badge R (1997) Sox genes find their feet. Curr Opin Genet Dev 7: 338–344. 9229109 10.1016/s0959-437x(97)80147-5

[66] Uwanogho D, Rex M, Cartwright EJ, Pearl G, Healy C, Scotting PJ, et al (1995) Embryonic expression of the chicken Sox2, Sox3 and Sox11 genes suggests an interactive role in neuronal development. Mech Dev 49: 23–36. 7748786 10.1016/0925-4773(94)00299-3

[67] Deaton AM, Bird A (2011) CpG islands and the regulation of transcription. Genes Dev 25: 1010–1022. doi: 10.1101/gad.2037511 21576262 10.1101/gad.2037511 PMC3093116

[68] Bernstein BE, Mikkelsen TS, Xie X, Kamal M, Huebert DJ, Cuff J, et al (2006) A bivalent chromatin structure marks key developmental genes in embryonic stem cells. Cell 125: 315–326. doi: 10.1016/j.cell.2006.02.041 16630819 10.1016/j.cell.2006.02.041

[69] Mohn F, Weber M, Rebhan M, Roloff TC, Richter J, Stadler MB, et al (2008) Lineage-specific polycomb targets and de novo DNA methylation define restriction and potential of neuronal progenitors. Mol Cell 30: 755–766. doi: 10.1016/j.molcel.2008.05.007 18514006 10.1016/j.molcel.2008.05.007

[70] Lauberth SM, Nakayama T, Wu X, Ferris AL, Tang Z, Hughes SH, et al (2013) H3K4me3 interactions with TAF3 regulate preinitiation complex assembly and selective gene activation. Cell 152: 1021–1036. doi: 10.1016/j.cell.2013.01.052 23452851 10.1016/j.cell.2013.01.052 PMC3588593

[71] Kurdistani SK, Tavazoie S, Grunstein M (2004) Mapping global histone acetylation patterns to gene expression. Cell 117: 721–733. doi: 10.1016/j.cell.2004.05.023 15186774 10.1016/j.cell.2004.05.023

[72] Kimura A, Matsubara K, Horikoshi M (2005) A decade of histone acetylation: marking eukaryotic chromosomes with specific codes. J Biochem 138: 647–662. doi: 10.1093/jb/mvi184 16428293 10.1093/jb/mvi184

[73] Rando OJ (2007) Global patterns of histone modifications. Curr Opin Genet Dev 17: 94–99. doi: 10.1016/j.gde.2007.02.006 17317148 10.1016/j.gde.2007.02.006

[74] Oldfield AJ, Yang P, Conway AE, Cinghu S, Freudenberg JM, Yellaboina S, et al (2014) Histone-fold domain protein NF-Y promotes chromatin accessibility for cell type-specific master transcription factors. Mol Cell 55: 708–722. doi: 10.1016/j.molcel.2014.07.005 25132174 10.1016/j.molcel.2014.07.005 PMC4157648

[75] Nardini M, Gnesutta N, Donati G, Gatta R, Forni C, Fossati A, et al (2013) Sequence-specific transcription factor NF-Y displays histone-like DNA binding and H2B-like ubiquitination. Cell 152: 132–143. doi: 10.1016/j.cell.2012.11.047 23332751 10.1016/j.cell.2012.11.047

[76] Milivojevic Milena N G, Kovacevic-Grujicic Natasa, Krstic Aleksandar, Mojsin Marija, Drakulic Danijela, Stevanovic Milena (2010) Involvement of ubiquitous and TALE transcription factors, as well as liganded RXRα, in the regulation of human SOX2 gene expression in the NT2/D1 embryonal carcinoma cell line. Archives of Biological Sciences, Belgrade 62: 199–210.

[77] Wiebe MS, Wilder PJ, Kelly D, Rizzino A (2000) Isolation, characterization, and differential expression of the murine Sox-2 promoter. Gene 246: 383–393. 10767561 10.1016/s0378-1119(00)00086-x

[78] Rogers CD, Harafuji N, Archer T, Cunningham DD, Casey ES (2009) Xenopus Sox3 activates sox2 and geminin and indirectly represses Xvent2 expression to induce neural progenitor formation at the expense of non-neural ectodermal derivatives. Mech Dev 126: 42–55. doi: 10.1016/j.mod.2008.10.005 18992330 10.1016/j.mod.2008.10.005 PMC2700551

[79] Tomioka M, Nishimoto M, Miyagi S, Katayanagi T, Fukui N, Niwa H, et al (2002) Identification of Sox-2 regulatory region which is under the control of Oct-3/4-Sox-2 complex. Nucleic Acids Res 30: 3202–3213. 12136102 10.1093/nar/gkf435 PMC135755

[80] Uchikawa M, Ishida Y, Takemoto T, Kamachi Y, Kondoh H (2003) Functional analysis of chicken Sox2 enhancers highlights an array of diverse regulatory elements that are conserved in mammals. Dev Cell 4: 509–519. 12689590 10.1016/s1534-5807(03)00088-1

[81] Mojsin M, Topalovic V, Vicentic JM, Schwirtlich M, Stanisavljevic D, Drakulic D, et al (2015) Crosstalk between SOXB1 proteins and WNT/beta-catenin signaling in NT2/D1 cells. Histochem Cell Biol 144: 429–441. doi: 10.1007/s00418-015-1352-0 26239426 10.1007/s00418-015-1352-0

[82] Niwa H, Ogawa K, Shimosato D, Adachi K (2009) A parallel circuit of LIF signalling pathways maintains pluripotency of mouse ES cells. Nature 460: 118–122. doi: 10.1038/nature08113 19571885 10.1038/nature08113

[83] Stavridis MP, Lunn JS, Collins BJ, Storey KG (2007) A discrete period of FGF-induced Erk1/2 signalling is required for vertebrate neural specification. Development 134: 2889–2894. doi: 10.1242/dev.02858 17660197 10.1242/dev.02858

[84] Takahashi K, Murakami M, Yamanaka S (2005) Role of the phosphoinositide 3-kinase pathway in mouse embryonic stem (ES) cells. Biochem Soc Trans 33: 1522–1525. doi: 10.1042/BST20051522 16246160 10.1042/BST0331522

[85] Armstrong L, Hughes O, Yung S, Hyslop L, Stewart R, Wappler I, et al (2006) The role of PI3K/AKT, MAPK/ERK and NFkappabeta signalling in the maintenance of human embryonic stem cell pluripotency and viability highlighted by transcriptional profiling and functional analysis. Hum Mol Genet 15: 1894–1913. doi: 10.1093/hmg/ddl112 16644866 10.1093/hmg/ddl112

[86] Smith JR, Vallier L, Lupo G, Alexander M, Harris WA, Pedersen RA (2008) Inhibition of Activin/Nodal signaling promotes specification of human embryonic stem cells into neuroectoderm. Dev Biol 313: 107–117. doi: 10.1016/j.ydbio.2007.10.003 18022151 10.1016/j.ydbio.2007.10.003

[87] Abello G, Khatri S, Radosevic M, Scotting PJ, Giraldez F, Alsina B (2010) Independent regulation of Sox3 and Lmx1b by FGF and BMP signaling influences the neurogenic and non-neurogenic domains in the chick otic placode. Dev Biol 339: 166–178. doi: 10.1016/j.ydbio.2009.12.027 20043898 10.1016/j.ydbio.2009.12.027

[88] Tay Y, Zhang J, Thomson AM, Lim B, Rigoutsos I (2008) MicroRNAs to Nanog, Oct4 and Sox2 coding regions modulate embryonic stem cell differentiation. Nature 455: 1124–1128. doi: 10.1038/nature07299 18806776 10.1038/nature07299

[89] Xu N, Papagiannakopoulos T, Pan G, Thomson JA, Kosik KS (2009) MicroRNA-145 regulates OCT4, SOX2, and KLF4 and represses pluripotency in human embryonic stem cells. Cell 137: 647–658. doi: 10.1016/j.cell.2009.02.038 19409607 10.1016/j.cell.2009.02.038

[90] Amaral PP, Neyt C, Wilkins SJ, Askarian-Amiri ME, Sunkin SM, Perkins AC, et al (2009) Complex architecture and regulated expression of the Sox2ot locus during vertebrate development. RNA 15: 2013–2027. doi: 10.1261/rna.1705309 19767420 10.1261/rna.1705309 PMC2764477

[91] Fei T, Xia K, Li Z, Zhou B, Zhu S, Chen H, et al (2010) Genome-wide mapping of SMAD target genes reveals the role of BMP signaling in embryonic stem cell fate determination. Genome Res 20: 36–44. doi: 10.1101/gr.092114.109 19926752 10.1101/gr.092114.109 PMC2798829

[92] Chambers SM, Fasano CA, Papapetrou EP, Tomishima M, Sadelain M, Studer L (2009) Highly efficient neural conversion of human ES and iPS cells by dual inhibition of SMAD signaling. Nat Biotechnol 27: 275–280. doi: 10.1038/nbt.1529 19252484 10.1038/nbt.1529 PMC2756723

