# Supplementary material for: Epigenetic regulation of human SOX3 gene expression during early phases of neural differentiation of NT2/D1 cells
Source: PLoS One. 2017 Sep 8;12(9):e0184099. doi: 10.1371/journal.pone.0184099 (PMC5590877; doi:10.1371/journal.pone.0184099)
Supplement: S1 Fig — Raw data were retrieved from http://www.roadmapepigenomics.org/ and converted in bigwig files through Galaxy tool followed by the visualization in UCSC genome browser. (PPTX) [file pone.0184099.s001.pptx]

## Slide 1
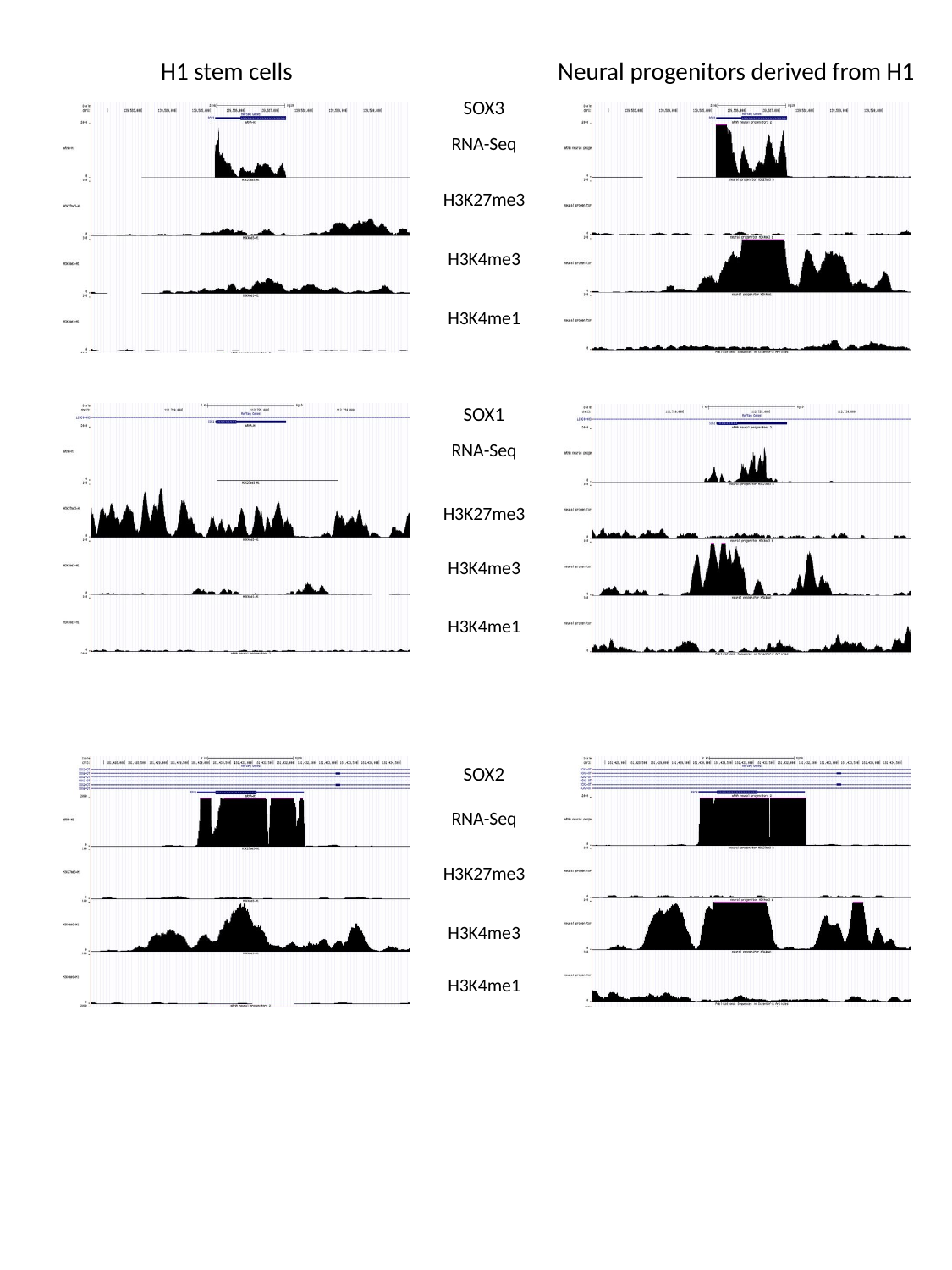

H1 stem cells
Neural progenitors derived from H1
SOX3
RNA-Seq
H3K27me3
H3K4me3
H3K4me1
SOX1
RNA-Seq
H3K27me3
H3K4me3
H3K4me1
SOX2
RNA-Seq
H3K27me3
H3K4me3
H3K4me1
